# A One Health study of Klebsiella pneumoniae species complex plasmids shows a highly diverse and ecologically adaptable plasmidome

**DOI:** 10.1099/mgen.0.001629

**Published:** 2026-02-19

**Authors:** Mia A. Winkler, Marit A. K. Hetland, Håkon Pedersen Kaspersen, Ragna-Johanne Bakksjø, Eva Bernhoff, Aasmund Fostervold, Jane Hawkey, Bjørn-Tore Lunestad, Nachiket P. Marathe, Niclas Raffelsberger, Ørjan Samuelsen, Marianne Sunde, Arnfinn Sundsfjord, Margaret M. C. Lam, Iren H. Löhr

**Affiliations:** 1 Department of Medical Biology, Faculty of Health Sciences, UiT The Arctic University of Norway, Tromsø, Norway; 2 Department of Medical Microbiology, Stavanger University Hospital, Stavanger, Norway; 3 Department of Biological Sciences, Faculty of Science and Technology, University of Bergen, Bergen, Norway; 4 Research Section Food Safety and Animal Health, Department of Animal Health and Food Safety, Norwegian Veterinary Institute, Oslo, Norway; 5 Department of Clinical Science, Faculty of Medicine, University of Bergen, Bergen, Norway; 6 Department of Infectious Diseases, Central Clinical School, Monash University, Melbourne, Australia; 7 Centre to Impact AMR, Monash University, Melbourne, Australia; 8 Institute of Marine Research, Bergen, Norway; 9 Department of Biological Sciences, University of Bergen, Bergen, Norway; 10 Department of Microbiology and Infection Control, University Hospital of North Norway, Tromsø, Norway; 11 Norwegian Centre for Detection of Antimicrobial Resistance, Department of Microbiology and Infection Control, University Hospital of North Norway, Tromsø, Norway; 12 Section for Bacteriology, Department for Analysis and Diagnostics, Norwegian Veterinary Institute, Ås, Norway

**Keywords:** antimicrobial resistance, *Klebsiella pneumoniae*, one health, plasmids

## Abstract

Plasmids play a pivotal role in the horizontal gene transfer (HGT) of antimicrobial resistance (AMR) and virulence determinants among bacteria. Members of the *Klebsiella pneumoniae* species complex (KpSC) can colonize humans, animals and various environments and frequently cause nosocomial and community-acquired infections in humans. While plasmid-borne AMR genes are prevalent in clinical strains, the diversity, distribution and association of plasmids encoding AMR and virulence across ecological niches remain poorly characterized. Understanding the traits governing successful plasmid transmission within and between ecological niches is critical for developing effective AMR prevention strategies. Here, we identify ecological and structural factors shaping plasmid persistence and dissemination. We analysed the plasmidome (i.e. total genetic content attributable to plasmids) of 578 whole-genome sequenced KpSC isolates collected in Norway between 2001 and 2020 from human (*n*=453), animal (*n*=102) and marine (*n*=23) sources. Plasmids from complete hybrid assemblies were annotated and clustered to evaluate the plasmid diversity and content across niches. Additionally, the representativeness of this plasmid collection was determined by clustering with a global collection of 8,656 circularized KpSC plasmids. In total, 1,415 circularized plasmids were identified and grouped according to rearrangement distance using Pling, resulting in 130 clusters (≥2 plasmids each), of which 36% (*n*=47) contained plasmids from more than one niche. The plasmids exhibited significant diversity, as 37% (*n*=524) remained singletons after clustering. AMR and virulence genes existed across diverse clusters and singletons but predominantly resided on 120–250 kbp conjugative or mobilizable plasmids harbouring various transposable elements. Human isolates carried higher overall plasmid burdens and harboured most AMR-encoding plasmids, while animal isolates were significantly enriched for virulence plasmids (*P*<0.001), largely due to *iuc*3 plasmids in pigs. Plasmids from human, animal and marine isolates formed shared genetic clusters spanning ecological boundaries, revealing the existence of widely distributed backbones already primed for AMR gene acquisition. The extensive diversity of KpSC plasmids highlights the dynamic nature of plasmid evolution, driven by HGT and selective pressures. The presence of variable clusters, marked by high genetic diversity, indicates a dynamic plasmidome capable of rapid adaptation to environmental pressures through the acquisition and rearrangement of accessory genes.

**Impact Statement**

*Klebsiella pneumoniae* is a global opportunistic pathogen and a major cause of nosocomial and community-acquired infections. Here, we present a large, nationwide analysis of plasmid diversity in *K. pneumoniae* species complex (KpSC) isolates collected from human, animal and marine sources in Norway. Our results reveal extensive plasmid diversity, with many plasmid backbones shared across ecological niches and substantial overlap between Norwegian and globally distributed antimicrobial resistance (AMR) plasmids. Notably, these plasmid backbones were identified even in a low-AMR setting, suggesting their persistence and potential for gene acquisition. This work demonstrates the dynamic and interconnected nature of the KpSC plasmidome and underscores the importance of incorporating plasmid surveillance, in addition to strain-based monitoring, in future One Health strategies to track and limit the spread of AMR and virulence traits.

## Data Summary

The Illumina and Oxford Nanopore Technologies reads for all isolates are available under project PRJEB74192 on the European Nucleotide Archive. The hybrid-assembled genomes have been deposited in GenBank. See Table S1 for metadata and genotyping results of the Norwegian dataset and Table S2 for accessions and genotyping results of the global dataset.

## Introduction

*Klebsiella pneumoniae* is a frequent cause of nosocomial and community-acquired infections [1,3]. Its ability to proliferate in humans, animals and various terrestrial and marine environments underscores its adaptability and public health importance [1,4,6]. The *K. pneumoniae* species complex (KpSC) encompasses seven closely related subspecies that can be further divided into sublineages (SLs) and sequence types (STs) based on genetic similarity [2,7, 8]. Clones associated with multidrug resistance (MDR) frequently harbour acquired antimicrobial resistance (AMR) genes that encode extended-spectrum *β*-lactamases (ESBLs) and carbapenemases, the majority of which are plasmid-borne and spread through horizontal gene transfer (HGT) [1,3, 9]. Hypervirulence-associated (HV) clones typically harbour genes encoding siderophores, aerobactin and salmochelin, and hypermucoidy co-residing on distinct plasmids [1,9, 10].

Plasmids serve as the primary vectors facilitating the transfer of AMR, virulence and heavy metal resistance (HMR) genes amongst bacteria via HGT [11,14]. KpSC isolates exhibit a diverse assortment of plasmids and often harbour multiple plasmids per isolate. The flexibility of the KpSC accessory genome reflects its promiscuity and capacity to acquire plasmids from a wide variety of species, making KpSC members efficient conduits for HGT. This adaptability positions KpSC as a reservoir for plasmids, facilitating recombination events amongst plasmids, which may in turn promote the spread of clinically relevant genes [1,11]. Consequently, understanding the KpSC plasmidome is necessary for the development of strategies aimed at curtailing the spread of AMR and virulence. Despite the central role of plasmids and other mobile genetic elements, the factors enabling their successful spread and persistence in KpSC across SLs and ecological niches remain poorly understood. Recent advances in long-read sequencing technologies and hybrid assemblies now enable detailed analyses of complete plasmid sequences, including structural variation and SNPs, providing opportunities to address this gap [15,17].

While our previous genome-wide One Health study [6] identified limited strain-sharing across niches, plasmid-level relationships remain unexplored. Here, we demonstrate that cross-niche gene flow in the KpSC is largely associated with plasmid connectivity rather than chromosomal overlap, providing new insight into the ecological and structural factors underlying plasmid persistence. We utilized a comprehensive collection of 1,415 closed plasmids from 578 hybrid-assembled genomes to examine the diversity of KpSC plasmids across ecological niches. The genomes were representative of a larger collection of 3,255 Norwegian KpSC isolates, sampled over two decades (2001–2020) from human, terrestrial animal (simply ‘animal’ hereafter) and marine sources [6,18,26]. By coupling long-read plasmid assemblies with niche metadata across the full KpSC, this study provides, to our knowledge, one of the first large-scale, high-resolution frameworks to characterize plasmid diversity and cross-niche persistence at a national scale. Using cross-sectoral genotyping and plasmid clustering, we further identified AMR and virulence determinants within shared plasmid clusters.

## Methods

### Sample selection and whole-genome sequencing

The isolates included in this study were selected as a subset of a larger collection of 3,255 KpSC isolates collected in Norway between 2001 and 2020 from human (*n*=2,656) [20,21, 26], animal (*n*=500) [19,24, 25] and marine (*n*=99) sources [18,22, 23]. All isolates were short-read sequenced on Illumina MiSeq or HiSeq 2500 platforms. Of these, 578 isolates (17.8% of 3,255) (*n*=453 human, *n*=102 animal, *n*=23 marine) were selected for additional long-read sequencing on Oxford Nanopore Technologies (ONT) platforms [6,27], which formed the full dataset for the present study. Isolates were selected based on KpSC diversity (i.e. to represent the diversity in the pangenome and at least one of each niche-overlapping SL) and the presence of clinically relevant genetic features, including AMR and virulence genes and plasmid replicon markers (see File S1 for details, available in the online Supplementary Material). Hybrid assembly was performed using both long-read-first and short-read-first approaches on all sequences to produce closed genomes and maximize plasmid recovery. Briefly, clinopore-nf v1.1 (https://github.com/HughCottingham/clinopore-nf) was used for the long-read-first approach to assemble genomes with Flye v2.9-b1768 [28] and polish with Medaka v1.5.0 (https://github.com/nanoporetech/medaka), Polypolish v0.5.0 [29] and POLCA v4.0.5 [30]. For the short-read-first approach, genomes were assembled with Unicycler v0.5.0 [31], using SPAdes v3.15.4 [32], and polished using the tools previously listed. Plasmid contigs from the two assemblers (<1 Mbp) were then clustered using Trycycler v0.5.3 [33] by a mash distance of ≤0.01. If a plasmid was found by only one assembler, we kept it; if a plasmid was detected by both assemblers and both plasmids were closed and of comparable length, we kept the plasmid from the Flye assembly. Unless otherwise specified, default parameters were used for all methods. Sequencing and assembly methods are described in detail in Hetland *et al.* [27].

### Plasmid annotation and genotyping

All genomes were annotated with Bakta v1.8.1 [34] with database v5.0 using the ‘complete’ flag (otherwise default settings) and homologous genes were clustered using Panaroo v1.3.3 [35]. The Bakta output was used to identify HMR genes (see File S1). Kleborate v2.4.0 [36] was used to identify species and ST, as well as virulence and AMR determinants on each replicon. MOB-typer v3.1.5 [37] was used to assign primary MOB cluster IDs, as well as predict plasmid mobility, GC content, MOB type and replicon markers. Transposable elements (TEs) were detected using Mobile Element Finder v1.1.2 [38]. SLs were defined previously [6] using the *Klebsiella* BIGSdb-Pasteur web tool (https://bigsdb.pasteur.fr/klebsiella/) to assign life identification number code prefixes based on core genome multilocus ST profiles [8].

The copy number of each plasmid contig (complete and incomplete, *n*=1,427) was estimated by mapping raw short and long reads to their corresponding closed genomes using Plassembler v1.8.0 [39] in ‘assembled’ mode using the `--skip_mash` flag and `--depth_filter` set to 0.05, otherwise default settings (database download 10 July 2025). Estimated copy number was assigned as follows: the value of `plasmid_copy_number_long` was used for (1) plasmids≥20 kbp and (2) plasmids<20 kbp and prepared with the ONT rapid kit; `plasmid_copy_number_short` was used for plasmids with a mean long read depth of zero and plasmids<20 kbp prepared with the ONT ligation kit. Values less than one were rounded up to one, and values greater than one were rounded to the nearest decimal. The plasmid load of an isolate was defined as the percentage of total genomic content attributable to plasmids, calculated by multiplying the length of each plasmid by its estimated copy number and dividing the total plasmid nucleotide content by the copy number-adjusted genome size. All plasmid contigs were used to determine the number of distinct plasmids per isolate and the total plasmid load of each isolate.

### Plasmid clustering and network analysis

#### Norwegian plasmids

The circularized plasmid sequences (*n*=1,415) from our collection were clustered with Pling v2.0 [17] using a batch size of 750 and default settings for other parameters. Briefly, plasmids within the containment distance threshold (i.e. the proportion of shared sequence between two plasmids relative to the smaller of the two, default=0.5) were grouped into communities. Plasmids were then further split into subcommunities (hereafter ‘clusters’) based on the maximum number of structural rearrangements separating two plasmid sequences (default=4), determined by the Double Cut and Join insertion–deletion (DCJ-indel) distance. Hub plasmids were defined as plasmids that were highly connected to other sparsely or non-connected plasmids or plasmid groups (default: node degree>10, neighbouring node’s edge density<0.2), which may erroneously connect unrelated plasmids, resulting in over-clustering [17] (see File S1 for more details). In addition to the hub plasmids detected by Pling, clusters were manually inspected. Additional plasmids were deemed hubs if they appeared to spuriously connect otherwise disparate plasmid groups within a cluster based on characteristics such as sequence length, AMR or virulence gene content, replicon markers or MOB classification. All identified hub plasmids were excluded from the final network visualization. In a small number of cases, Pling clusters that contained plasmids with distinct, non-overlapping length distributions and differing replicon or gene content were manually subdivided into separate clusters to reflect clearly distinct plasmid backbones. These subdivided clusters were labelled by appending a capital letter suffix to the original cluster identifier.

#### Global plasmids

To assess whether our collection of Norwegian plasmids was representative of the global distribution of KpSC plasmids, we retrieved publicly available complete genomes from NCBI’s RefSeq database on 11 December 2024. The search date range was from 1 January 2015 to 11 December 2024 and included all KpSC members. The 578 genomes analysed in this study were removed from the search to avoid duplicate sequences. All genomes were analysed using Kleborate v2.4.0 to confirm species assignment and identify AMR and/or virulence genes. Publicly available genomes were then excluded if: (1) the chromosome was not complete (circularized); (2) the species reported in the RefSeq database was not reported or was not in agreement with the species reported by Kleborate; (3) Kleborate reported a ‘weak’ species match; and/or (4) sample geographical location was not reported in the RefSeq database. Of the remaining genomes, only complete, circularized plasmid sequences were extracted (*n*=8,656) and combined with the plasmids from our dataset, resulting in *n*=10,071 plasmids from 65 countries in six world regions. These were clustered with Pling using the same parameters as described above to assess the representativeness of our Norwegian KpSC plasmid collection.

### Statistical analysis

Statistical analyses were performed in R version 4.3.1 (16 June 2023); comparisons were made using Kruskal–Wallis (overall) and Mann–Whitney (pairwise) tests for range and chi-squared tests for proportions (overall and pairwise). *P* values<0.05 were considered statistically significant and reported as follows: *P<0.05, **P<0.01, ***P<0.001, ****P<0.0001, ns *P*≥0.05.

### Definitions

MDR plasmids: plasmids with a Kleborate num_resistance_classes value≥3; defined by presence of AMR genes and mutations identified by Kleborate.

## Results

The 578 genomes represented the human (78.4%, *n*=453/578), animal (17.6%, *n*=102) or marine niche (4.0%, *n*=23), and the majority belonged to *K. pneumoniae sensu stricto*, hereafter *K. pneumoniae* (85.1%, *n*=492), followed by *Klebsiella variicola* subsp. *variicola*, hereafter *K. variicola* (11.9%, *n*=69), *Klebsiella quasipneumoniae* subsp. *similipneumoniae* (1.6%, *n*=9), *K. quasipneumoniae* subsp. *quasipneumoniae* (1.2%, *n*=7) and a single *Klebsiella quasivariicola* isolate (0.2%). The 578 genomes belonged to 291 SLs, the majority of which were represented by a single isolate (72.2%, *n*=210). The most prevalent SLs in this subset were SL17 (*n*=24, 4.2%), SL3010 and SL37 (each *n*=23, 4.0%), SL107 (*n*=21, 3.6%), SL35 (*n*=16, 2.8%), SL45 (*n*=15, 2.6%) and SL258 (*n*=11, 1.9%), which largely reflected the SL prevalence in the broader collection [6].

A total of 568/578 (98.3%) whole-genome sequenced KpSC isolates in this study were fully closed (i.e. all chromosomes and plasmids, if present, were circularized). One genome contained an unclosed chromosome and one unclosed plasmid, and nine genomes contained at least one unclosed plasmid, for a total of eleven unclosed plasmids. Of the 578 genomes, 88.9% (*n*=514) contained closed plasmids. In total, 1,427 plasmids were present in the genome collection: 1,415 complete and circularized, 1 complete and linear as confirmed by comparing with a set of previously defined linear plasmids [40] and 11 were not fully closed. All plasmid sequences (*n*=1,427) were used to assess plasmid burden across niches, while the 1,415 circularized plasmids were used in plasmid clustering and characterization.

### Plasmid load varied by niche

**Fig. 1.**
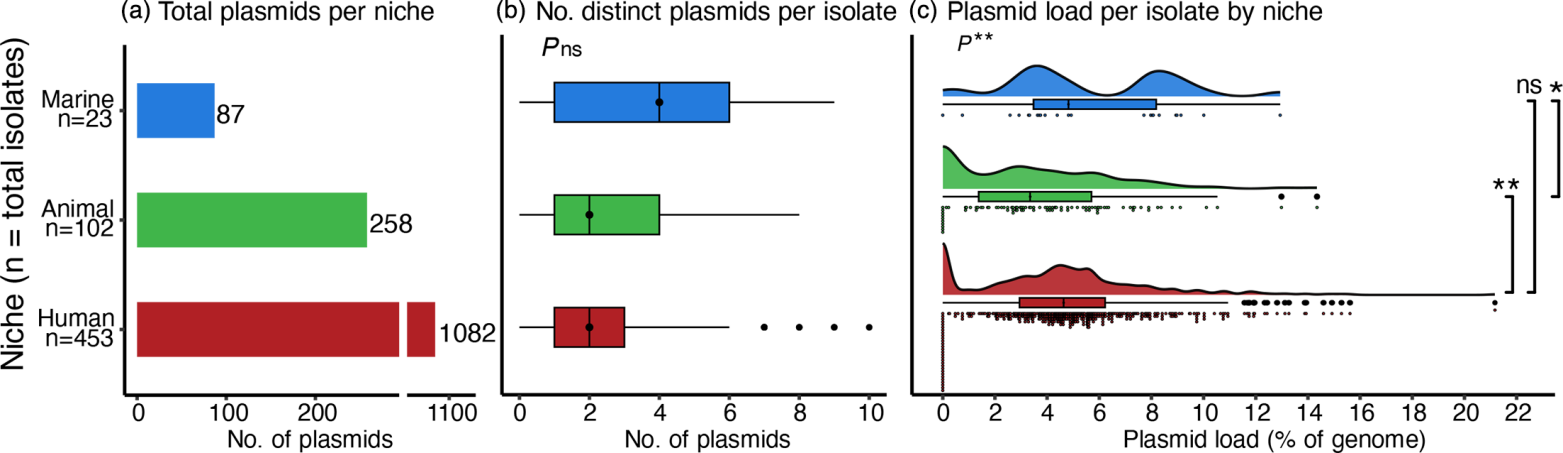
Plasmid distribution across niches. (**a**) Total number of plasmids recovered per niche. (**b**) Distribution of the number of distinct plasmid sequences per isolate within each niche, black vertical line indicates median value. (**c**) Distribution of estimated isolate plasmid load within each niche. Statistical comparisons were performed using Kruskal–Wallis (overall) and Mann–Whitney (pairwise). Significance is denoted as follows: *P<0.05, **P<0.01, ns *P*≥0.05.

The distribution of plasmids closely reflected the number of isolates from each niche: the majority (75.8%, *n*=1,082/1,427) were present in isolates from the human niche, followed by the animal (18.1%, *n*=258/1,427) and marine (6.1%, *n*=87/1,427) niches (Fig. 1a). The number of distinct plasmid sequences per genome (i.e. one representative of each, regardless of estimated copy number) ranged from 0 to 10 overall, with marine isolates typically having a higher number of plasmids per isolate (0–9 plasmids, median=4) than those from the human (0–10 plasmids, median=2) and animal (0–8 plasmids, median=2) niches. However, the differences were not significant (Kruskal–Wallis, *P*>0.05) (Fig. 1b).

While the number of distinct plasmids per isolate did not vary significantly by niche, the overall plasmid burden did. Plasmid load (i.e. percentage of each genome consisting of plasmid sequence when accounting for estimated copy number, see Methods) ranged from 0 to 21.2% (median 5.5%) overall. It was significantly lower in the animal niche (0–14.3%, median 3.4%) compared to the human (0–21.2%, median 4.6%) (*P*<0.01) and marine (0–12.9%, median 4.8%) (*P*<0.05) niches (Fig. 1c).

Most plasmids were found in *K. pneumoniae* (87.0%, *n*=1,241/1,427 plasmids among 447 isolates), followed by *K. variicola* (10.2%, *n*=146/1,427 plasmids among 55 isolates), *K. quasipneumoniae* subsp. *similipneumoniae* and *quasipneumoniae* (1.1%, *n*=16/1,427 plasmids each among 7 and 4 isolates, respectively) (Fig. S1A), and a single *K. quasivariicola* isolate harboured 0.6% of all plasmids (*n*=8/1,427). While both the number of distinct plasmids per isolate (Fig. S1B) and estimated plasmid load differed between species (Fig. S1C), the only statistically significant difference was the higher plasmid load in *K. pneumoniae* (0–21.2%, median 4.6%) versus *K. quasipneumoniae* subsp. *similipneumoniae* (0–5.1%, median 2.1%, *P*<0.05).

### Plasmids were diverse within and across niches

**Fig. 2.**
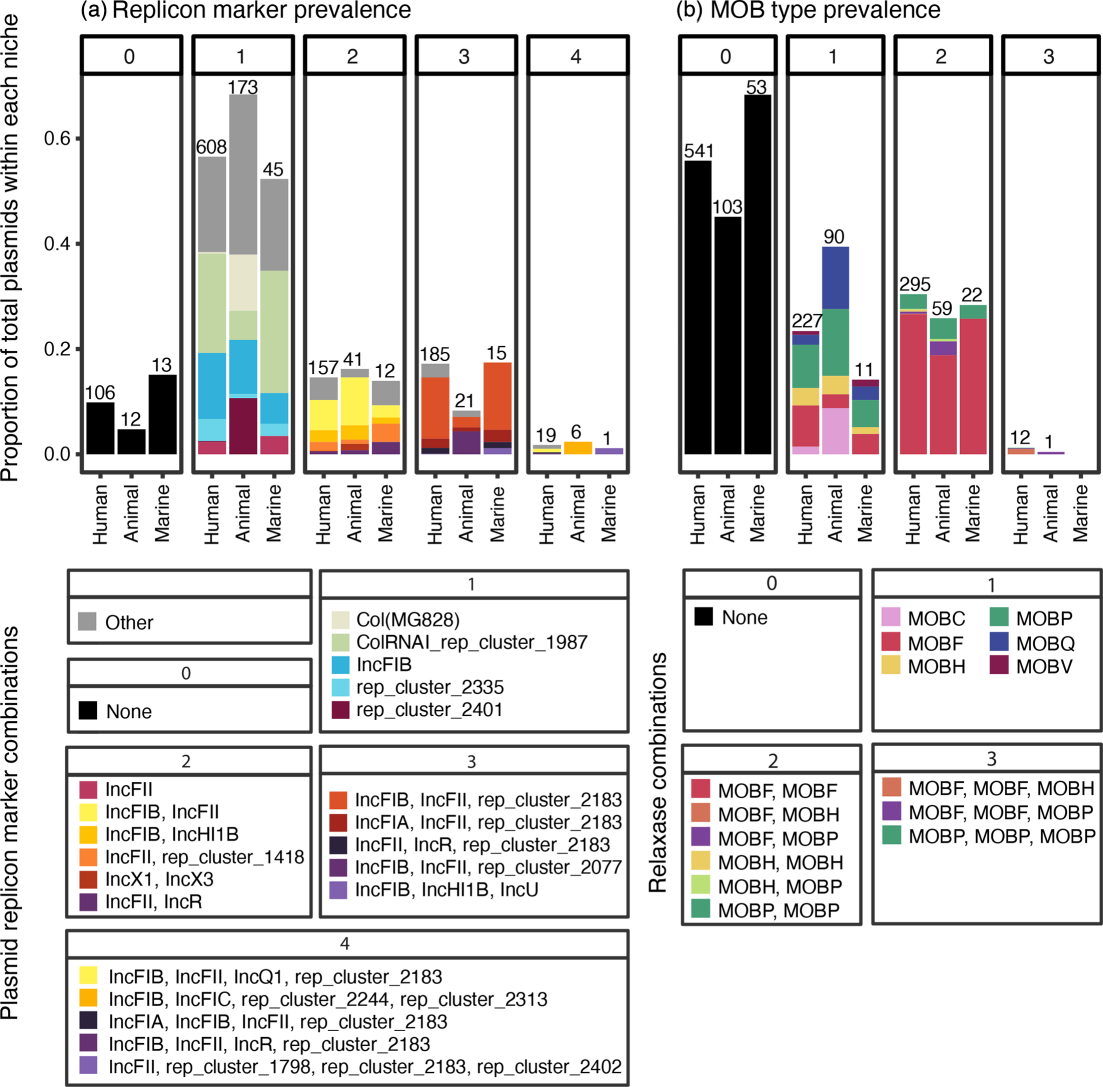
Replicon and MOB type distribution across niches. (**a**) Replicon marker prevalence by niche. Facet numbers indicate the number of markers per plasmid. Bars indicate the proportion of plasmids within each niche harbouring different replicon markers/combinations; total number of plasmids with specified number of replicon markers per niche above each bar. The three most frequent marker types for each niche/replicon number combination are shown; the rest are categorized as ‘Other’. (**b**) MOB type prevalence by niche. Facet numbers indicate the number of relaxases per plasmid. Bars indicate the proportion of plasmids within each niche harbouring different relaxase combinations corresponding to the number of relaxases per plasmid; total number of plasmids with specified number of relaxases per niche above each bar.

The 1,415 circularized plasmids were used to characterize, cluster and compare plasmids across the niches. Among the circularized plasmids, a total of 2,013 plasmid replicons (69 distinct replicon types) were identified (Table S1). Plasmids contained 0–4 replicon markers per plasmid (median=1) with slight variation across niches (Fig. 2a). The majority of plasmids harboured a single replicon marker (*n*=826/1,415, 58.4%), the most prevalent were ColRNAI_rep_cluster_1987 in both the human and marine niches (203 and 20 plasmids, respectively) and Col (MG828) and rep_cluster_2401 in the animal niche (in 27 plasmids each). In all niches, the second most common replicon on single replicon plasmids was IncFIB (135, 26 and 5 plasmids across human, animal and marine niches, respectively). Plasmids harbouring two replicon markers (14.8%, *n*=210/1,415) comprised the second largest portion of plasmids, with IncFIB/IncFII being the most frequent combination in the human and animal niches (62 and 23 plasmids, respectively) and IncFIB/rep_cluster_1418 in the marine niche (3 plasmids). The remaining plasmids harboured zero, three or four replicon markers (*n*=131, *n*=221, *n*=26, respectively).

Most plasmids (59.2%, *n*=837/1,415) were predicted to be transmissible (*n*=485 conjugative, *n*=352 mobilizable), while 40.8% (*n*=578/1,415) were predicted to be non-mobilizable. In total, 1,119 relaxases belonging to MOBC, MOBF, MOBH, MOBP, MOBQ and MOBV types were identified. Plasmids carried 0–3 relaxases (median=1) in various combinations. Nearly half of plasmids did not harbour a known relaxase (49.2%, *n*=697/1,415). Of these, 17.1% (*n*=119/697) were predicted to be mobilizable based on the presence of an origin of transfer. Almost one quarter of all plasmids harboured a single relaxase (23.2%, *n*=328/1,415) and corresponded to the largest portion of plasmids from the animal niche (35.6%, *n*=90/253); they most frequently encoded MOBP in all niches. Plasmids carrying two relaxases were the largest groups in the human and marine niches (27.4 and 25.6%, respectively); the most common pairings were MOBF/MOBF, followed by MOBP/MOBP in all niches (Fig. 2b).

One plasmid harboured more than four replicon markers; this was likely misassembled, as it was much larger than other plasmids assigned to the same cluster (>149 kbp) and contained exact combinations of replicon and relaxase markers observed in other plasmids roughly half its size assigned to the same grouping in the cluster analysis (below); it was therefore reclassified as a hub plasmid in the cluster analysis and excluded from replicon marker and relaxase visualizations and calculations.

### Clinically relevant plasmid-borne features varied across niches

Of the 514 genomes that carried plasmids, 28.6% (*n*=147/514) carried ≥1 plasmid encoding AMR genes, corresponding to a total of 181 AMR-encoding plasmids (12.8% of circularized plasmids). A total of 103 AMR-encoding plasmids had a resistance score of 0 as reported by Kleborate, corresponding to the presence of AMR genes but excluding ESBLs and carbapenemases; 73 plasmids had a resistance score of 1 (encoded ESBL without carbapenemase), the majority of which encoded *bla*_CTX-M-15_ (61.7%, *n*=45/73); 5 plasmids had a resistance score of 2 (encoded carbapenemase: *n*=2 *bla*_KPC-2_, *n*=2 *bla*_OXA-48_, *n*=1 *bla*_KPC-3_). Virulence genes or loci encoding aerobactin, salmochelin, hypermucoidy or yersiniabactin (*iuc*, *iro*, *rmpADC*, *rmpA2* or *ybt*, excluding truncated or incomplete hits) were detected on plasmids in 14.8% (*n*=76/514) of plasmid-containing genomes, across 76 plasmids (5.4% of circularized plasmids). Kleborate virulence scores for these plasmids ranged from 0 to 3; note that only yersiniabactin (*ybt*) and aerobactin (*iuc*) contribute to this score. Two virulence plasmids encoded only salmochelin (*iro*5) and therefore had a virulence score of 0, and seven plasmids encoded only yersiniabactin (virulence score of 1). The remaining 67 plasmids had a virulence score of 3 and encoded at least aerobactin, including 39 encoding aerobactin alone, 11 encoding aerobactin and salmochelin, 2 encoding aerobactin and hypermucoidy and 15 encoding aerobactin, salmochelin and hypermucoidy. The *iuc*3 locus was most prevalent amongst the aerobactin plasmids (49.3%, *n*=33/67), followed by *iuc*1 (*n*=15), *iuc*2 (*n*=10), *iuc*5 (*n*=8) and one KpVP-2/*iuc*2A hybrid plasmid. HMR genes were more frequently observed, found in 65.3% (*n*=336/514) of genomes and encoded by 377 plasmids (26.6%). Silver (*silABCERS*), copper (*pcoABCDRS*) and arsenic (*arsAD*, *arsBCR* or *arsABCDR*) resistance were most frequently detected overall (*n*=270, 266 and 228, respectively), and the combination of all three was also the most common multi-operon configuration amongst HMR plasmids (27.1%, *n*=102/377).

**Fig. 3.**
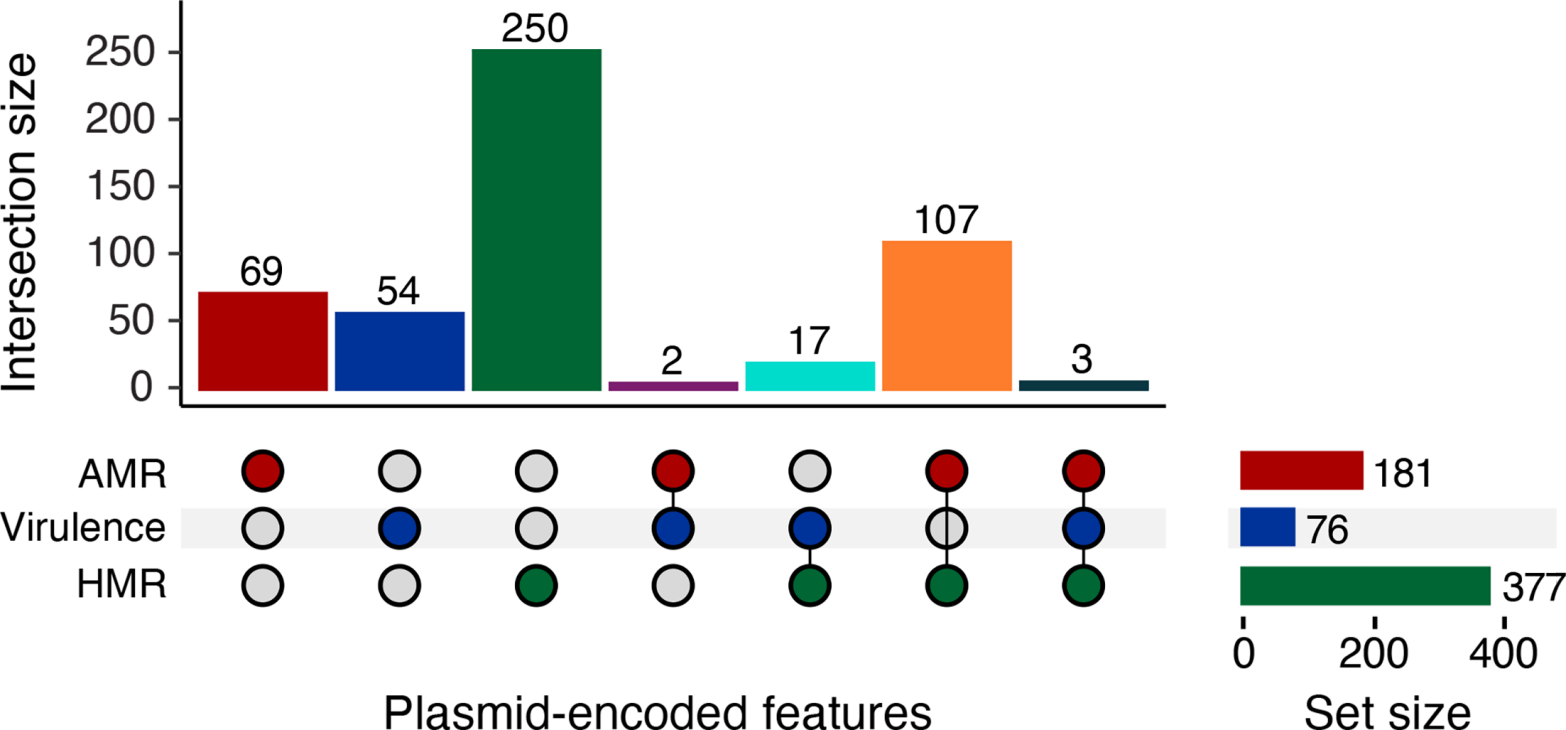
Overlap of clinically relevant features across plasmids. Upset plot showing the overlap of clinically relevant features harboured across n=502/1,415 plasmids. AMR genes in red, virulence factors in blue and HMR genes in green.

A subset of genomes (28.0%, *n*=144/514) harboured multiple plasmid-encoded clinically relevant traits, with features either carried on the same or on separate plasmids. In total, 117 genomes (22.8%) encoded AMR and HMR genes; 11 genomes (2.1%) carried HMR and other virulence genes; 4 genomes (0.8%) carried AMR and virulence genes; and 11 genomes (2.1%) harboured genes conferring AMR, HMR and other virulence factors (Fig. S2). Many genomes with several clinically relevant features could be explained by the presence of single plasmids harbouring these traits. The most frequent combination was AMR and HMR genes, co-encoded on 7.6% (*n*=107/1,415) of plasmids. HMR and other virulence genes were detected on 1.2% (*n*=17/1,415) of plasmids. Five plasmids (3.5%) carried both AMR and virulence genes, three of which also encoded HMR (Fig. 3). Of these, only one plasmid (belonging to an SL15 isolate from human blood) encoded an ESBL (*bla*_CTX-M-15_) together with the *iuc*1 locus and mercury resistance genes (*merACDEPRT*) (Fig. S3).

The convergent AMR and virulence plasmids (*n*=5) were only observed among human infection isolates belonging to SL15 (*n*=2), SL641 (*n*=1), SL846 (*n*=1) and SL881 (*n*=1) and harboured at least an IncFIB replicon. Four were predicted to be conjugative and encoded MOBF (*n*=2), MOBH (*n*=1) or MOBF/MOBP (*n*=1) relaxases, and one plasmid lacked a known relaxase and was predicted to be non-mobilizable. Of the 181 plasmids carrying AMR genes, 112 conferred MDR. Plasmids encoding AMR were present in all niches but primarily associated with the human niche (*n*=163/1,076, *n*=12/253 and *n*=6/86 plasmids in the human, animal and marine niches, respectively). The *iuc*3, *iuc*5 and *iro*5 virulence loci were enriched in the animal niche (*P*<0.05), with *iuc*3 specifically associated with pigs, as previously reported [24,41], and *iuc*5+*iro*5 loci linked to a previously reported SL290 clonal expansion in turkeys [25], while *iuc*1 was enriched in the human niche (*P*<0.05).

Overall, we observed high plasmid diversity in this collection, with sequence lengths ranging from 1.2 to 424 kbp (median=57 kbp); however, AMR genes were present across plasmids of a broad size range, 2.9–353 kbp (median=157 kbp), while virulence factors were typically encoded on 120–250 kbp (median=177 kbp) plasmids (Fig. S4).

AMR genes and/or virulence factors were observed on plasmids with a variety of replicon markers and MOB types (34 replicons, 5 MOB types). We observed the correlation of several AMR and virulence genes with particular replicon or MOB types (Fig. S5). Among the ESBL-encoding genes, *bla*_CTX-M_ variants (particularly *bla*_CTX-M-3_, *bla*_CTX-M-14_ and *bla*_CTX-M-15_) were most commonly found on MDR plasmids harbouring IncFIB and IncFII replicons and ≥1 MOBF relaxase. The siderophore-encoding loci (*iuc*, *iro*) and capsule regulators (*rmpA*, *rmpA2*) were also most frequently found on plasmids harbouring at least one IncFIB and one IncFII replicon and ≥1 MOBF relaxase. While IncFIB/IncFII and MOBF/MOBF plasmids were also common amongst plasmids lacking AMR genes and/or virulence loci, the majority of these plasmids harboured a ColRNAI_rep_cluster_1987 replicon and lacked a relaxase.

### TEs and plasmid mobility differed by niche and gene content

Both AMR genes and virulence factors primarily resided on conjugative plasmids containing 0–42 (median=12) TEs on AMR plasmids and 2–35 (median=6) TEs on virulence plasmids, with the exception of *iuc*1-encoding plasmids, which harboured more TEs on average compared to other virulence plasmids (12–35, median=15) and most were putatively non-mobilizable (*n*=11/15). Insertion sequences (ISs) were the dominant TE (Fig. S6A–D), accounting for 98.0% of TEs. Plasmids harbouring AMR genes exhibited significantly higher counts of IS elements (0–42, median=14) compared to those without AMR (0–47, median=0) (*P*<0.001) (Fig. S6B).

TE profiles also varied by ecological niche (Table S1). Plasmids from both human and marine sources contained significantly more IS elements (0–47, median=7; 0–37, median=2, respectively) compared to those from the animal niche (0–35, median=0) (*P*<0.01 and *P*<0.001, respectively). However, miniature inverted-repeat TEs were significantly enriched in the animal niche (6.32%, *n*=16/253 plasmids) compared to the human niche (1.49%, *n*=16/1,076 plasmids) (*P*<0.001). Unit transposons were more frequent in plasmids from the human niche (9.76%, *n*=105/1,076) compared to the animal niche (3.16%, *n*=8/253) (*P*<0.001) but not the marine niche (6.98%, *n*=6/86) (*P*>0.05).

### KpSC plasmid clustering showed high diversity

**Fig. 4.**
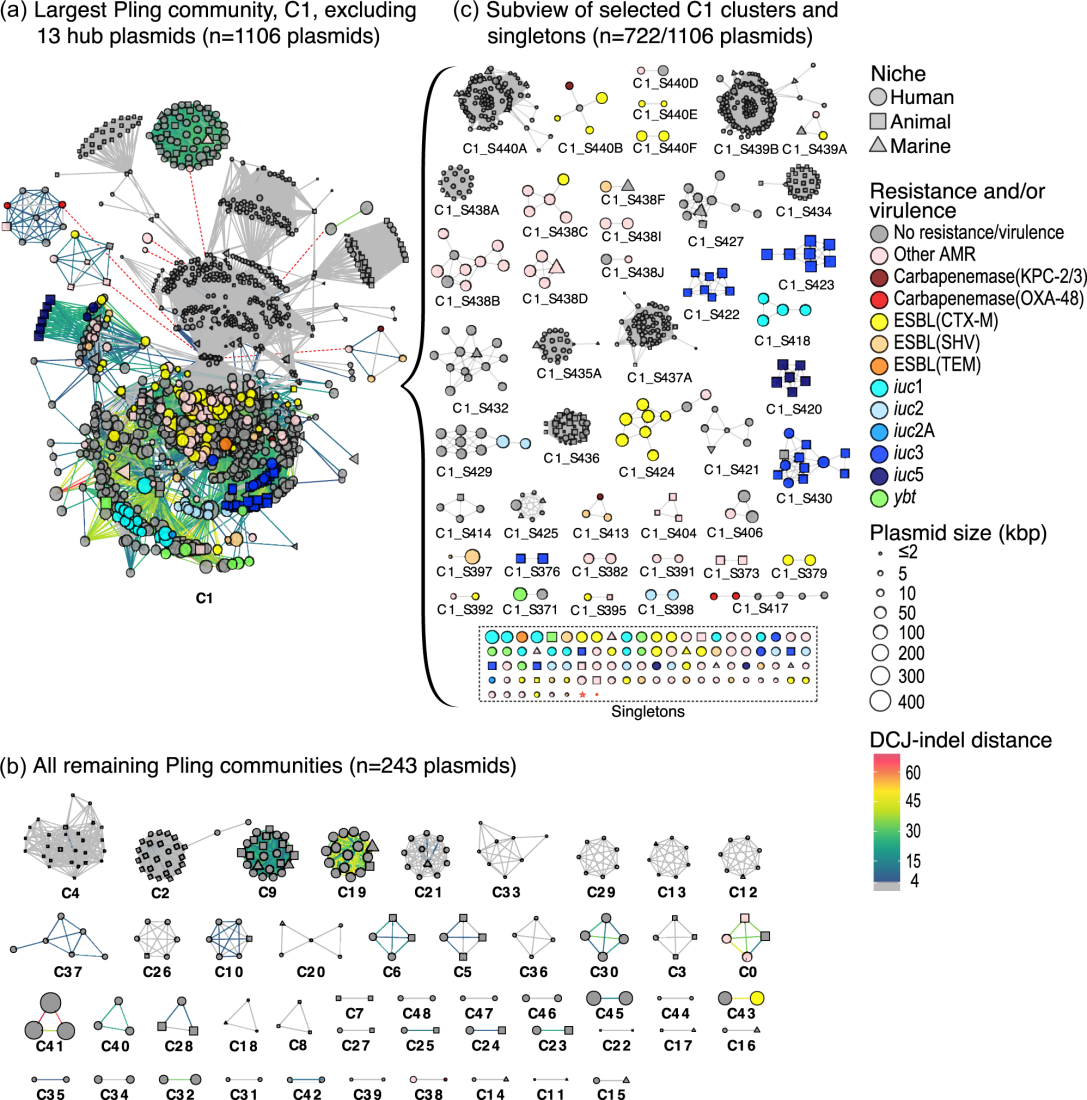
Pling network graph of *n*=1,415 circularized plasmids. (**a**) and (b) Plasmids belonging to *n*=49 Pling communities (*n*=1,349 plasmids), excluding 13 hub plasmids; red dotted lines indicate cluster connections that would have been made via hub plasmids. (**c**) Subview of selected clusters and singletons within community C1. Shown are clusters that (**i**) contain ≥1 AMR and/or virulence plasmid or (ii) comprise ≥4 plasmids derived from ≥2 niches and (iii) singletons that encode ≥1 AMR gene and/or virulence factor (shown in box). Node shape indicates isolate niche, node size indicates plasmid length in kbp and node colour represents clinically relevant plasmid-encoded features. Edge colour indicates DCJ-indel distance between plasmids within the same community, where grey indicates ≤4 rearrangements between sequences. Not shown: clusters containing <4 plasmids (*n*=84 clusters), hub plasmids (*n*=13) and singletons lacking clinically relevant genes (*n*=389 plasmids). For the complete Pling network, including hub plasmids and all singletons, see Fig. S7.

Clustering the circularized plasmids (*n*=1,415) with Pling (see details in Methods) resulted in 49 communities (i.e. plasmids within the containment distance threshold of 0.5). These were broken down further into 130 subcommunities (plasmids within 4 rearrangements of each other, hereafter referred to as ‘clusters’), 524 singletons and 13 hub plasmids (highly connected plasmids that lead to over-clustering). The plasmid collection exhibited considerable variation in size and content. The 49 communities ranged in size from 2 to 1,119 plasmids per community (median=3), encompassing 1,362 plasmids (Fig. 4). The largest community accounted for the vast majority of plasmids overall (79.1%, *n*=1,119/1,415), including 81.9% (*n*=429/524) of all singletons, and all hub plasmids. Excluding hub plasmids, a total of 878 plasmids were grouped into 130 distinct clusters, ranging in size from 2 to 116 plasmids per cluster (median=16). The 524 singleton plasmids accounted for 37.0% of the overall collection with a notable portion of those found in each of the human, animal and marine niches (40.5, 22.9 and 34.9% of each niche, respectively), demonstrating substantial plasmidome diversity. Pling detected ten hub plasmids and an additional three were identified upon inspection of the network clusters: ten were found in the human niche, of which two encoded ESBLs (*bla*_CTX-M-15_ and *bla*_SHV-2_); three hubs were from the marine niche, none of which encoded AMR or virulence. All hub plasmids were excluded from network visualization (Fig. 4; see Fig. S7 for a complete network including hubs and singletons).

To get a broad overview of plasmid distribution patterns across the KpSC population, we assessed if some SLs harboured a wider range of plasmid types than others and if some plasmid types had broader host (SL) ranges (Fig. S9).

Among the 514 plasmid-carrying isolates, 255 SLs were represented. Each SL carried between 1 and 53 (median=3, mean=4.29) unique plasmid types (including plasmid clusters and singletons). SLs with the greatest plasmid-type diversity were generally those with the largest number of isolates (Fig. S9), including SL3010, SL17, SL37, SL45 and SL35, which we previously identified as old, generalist clones with broad ecological ranges [6]. Notably, SL15 and SL258, both associated with nosocomial outbreaks [42], also carried a wide range of plasmid types.

Across the 130 plasmid clusters detected in the dataset (excluding hub plasmids), most spanned multiple SLs (*n*=102/130), while 28 clusters were restricted to a single SL. The number of SLs represented per plasmid cluster ranged from 1 to 63 (median=2, mean=4.39). The largest plasmid clusters tended to have the widest SL ranges (Fig. S9). Of the multi-SL clusters, 11 had plasmids present in ≥10 SLs, 8 of which were predominantly composed of small (<15 kbp, median<10 kbp, mean<10 kbp) plasmids, most of which were predicted to be mobilizable. The remaining three clusters consisted of: (i) plasmids ranging from 54.3 to 108.4 kbp (median=54.3 kbp, mean=58.9 kbp), all of which were non-mobilizable; (ii) 68.2–91.7 kbp plasmids (median=75.7 kbp, mean=77.3 kbp), most of which were mobilizable (92.3%, *n*=12/13); and (iii) 148.4–177.5 kbp plasmids (median=154.4 kbp, mean=155.2 kbp), most of which (91.2%, *n*=11/12) encoded *iuc*3, and all of which were predicted to be conjugative.

AMR and/or virulence genes were detected in 37 clusters, 132 singletons and 5 hub plasmids (*n*=252/1,415 plasmids); 28 clusters, 101 singletons and 5 hub plasmids contained at least one AMR plasmid (*n*=176/1,415 plasmids); and 9 clusters and 29 singletons contained plasmids encoding virulence factors (*n*=71/1,415 plasmids). Five singleton plasmids contained both AMR and virulence genes. All were found in human infection isolates of various SLs (SL641, *n*=1; SL846, *n*=1; SL881, *n*=1; and SL15, *n*=2). Four of the five plasmids encoded resistance to aminoglycosides; the fifth, found in SL881, encoded *iuc*3 and tetracycline resistance. The plasmid from SL846 encoded *iro*5, while the plasmid from SL641 encoded both *iuc*5 and *iro*5. The two plasmids from SL15 isolates encoded *iuc*1, and one of these plasmids also harboured *bla*_CTX-M-15_ and *bla*_SHV-5_.

### Within-cluster diversity

**Fig. 5.**
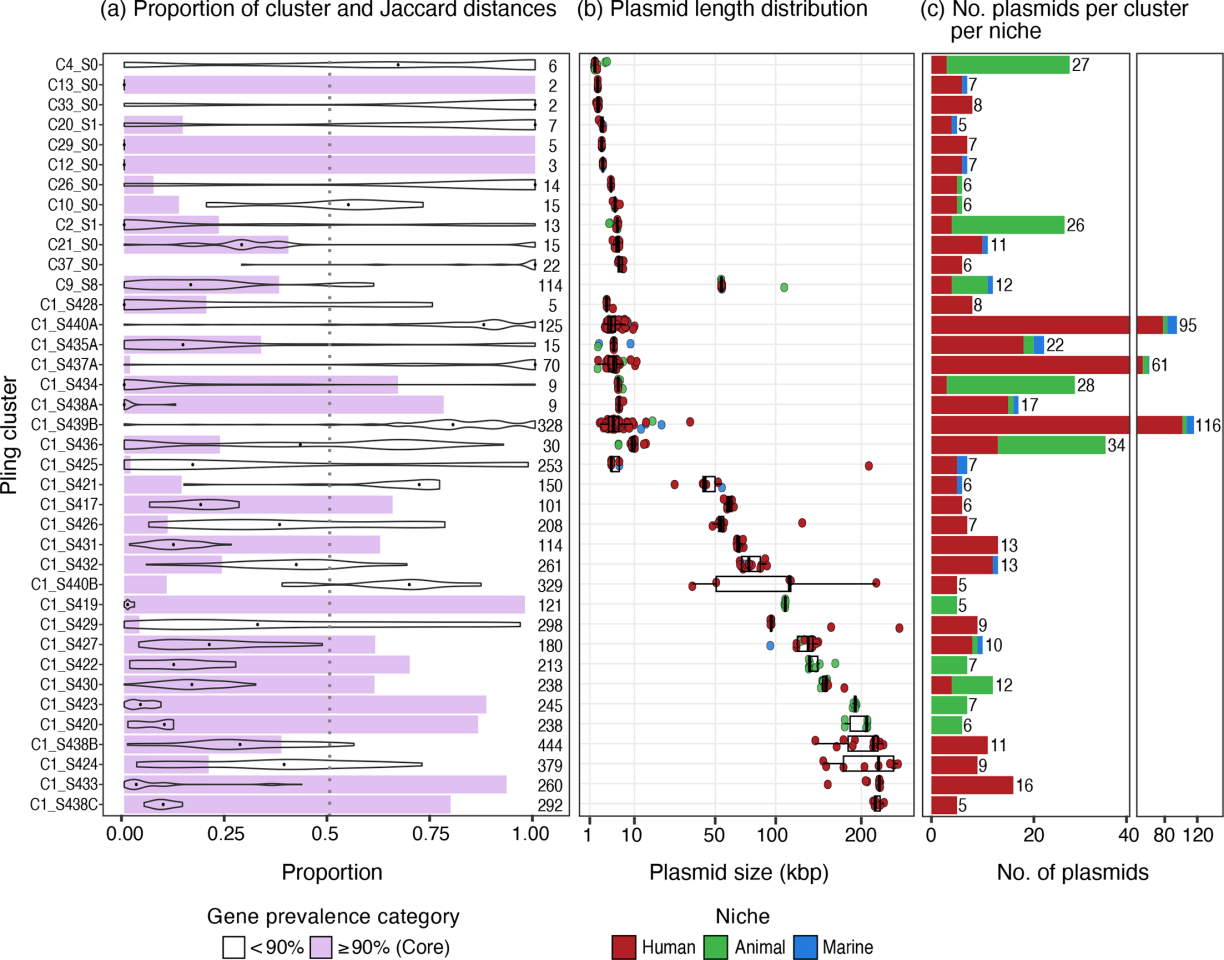
Within-cluster diversity. (**a**) Proportion of genes in selected clusters that were found in ≥90% of cluster plasmids (i.e. core genes relative to cluster, purple) and distribution of within-cluster pairwise Jaccard distances between plasmids; sum of the total number of Bakta-annotated genes per cluster next to each bar. (**b**) Distribution of plasmid lengths within each cluster (kbp), coloured according to isolate niche. (**c**) Number of plasmids per cluster from each of the human (red), animal (green) and marine (blue) niches. Not shown: clusters with <5 plasmids (*n*=92 clusters).

There were 9,711 annotated genes among the 1,415 plasmids. Within each cluster, we compared pairwise Jaccard distances and categorized genes as core (present in ≥90% of plasmids) or accessory (<90% of plasmids) (Fig. 5).

There were 38 clusters containing ≥5 plasmids, which accounted for 53.1% of plasmids (*n*=752/1,415). Five of these clusters lacked any core genes. These included four diverse clusters with plasmids from all three niches, including the two largest clusters (*n*=116 and *n*=95 plasmids). Interestingly, the fifth cluster lacking core genes contained plasmids from the human niche (*n*=6 plasmids) within a narrow length range (5.6–6.9 kbp).

Of 130 clusters (excluding hub plasmids), 47 (36.2%) included plasmids originating from multiple niches (15 human-animal, 22 human-marine, 3 animal-marine and 7 from all three niches), accounting for 42.9% (*n*=607/1,415) of plasmids overall (see Fig. 4). We have previously shown that within-niche strain-sharing occurred more frequently than strain-sharing events across ecological niches [6]. Because strain-sharing and plasmid-sharing clusters were defined using different criteria (isolates differing by ≤22 chromosomal SNPs versus belonging to the same Pling cluster), the data are not directly comparable. However, the greater number of plasmid-sharing Pling clusters relative to strain-sharing clusters suggests that plasmid-sharing occurred more frequently than strain-sharing both within and across niches (Fig. S8).

Some clusters appeared highly conserved; for example, clusters C1_S376, C1_S422, C1_S423 and C1_S430 comprised 28 *iuc*3-encoding plasmids (one of which was a truncated variant) predominantly from the animal niche (*n*=23 pig, *n*=1 dog), though C1_S430 also contained plasmids from the human niche (*n*=3 infection, *n*=1 carriage). Plasmids in these clusters were of similar length (134–202 kbp, median=156 kbp) and all harboured MOBFMOBF relaxases and IncFIB/IncFII replicon markers; however, they were distributed across diverse SLs, with the only overlaps occurring in SL35 (*n*=1 pig, *n*=1 human infection) and SL37 (*n*=3 pig, *n*=1 dog). Another conserved cluster, C1_S427, contained ten plasmids from all three niches (*n*=8 human, *n*=1 animal, *n*=1 marine), all lacking AMR or virulence genes and predicted non-mobilizable with a single IncFIB(K) replicon. Interestingly, the plasmid from the animal niche (turkey) carried the same HMR operons (*arsABCDR*, *pcoABCDRS*, *silABCERS*) as six of the plasmids from the human niche (five from human infections, one from carriage). While plasmids from the turkey isolate and two of the human infection isolates were harboured by the same SL (SL152), the remaining four plasmids from human infection and one carriage isolate were each harboured by distinct SLs.

Other clusters appeared far more diverse at first glance, such as C1_S438 and C1_S440; however, further inspection and removal of three additional hub plasmids resulted in slightly more homogenous clusters. Clusters C1_S438A-B initially belonged to the same group (C1_S438) but were further divided based on plasmid length. C1_S438B was a highly conserved cluster consisting of 17 5.9–6.9 kbp plasmids from three niches (*n*=15 human, *n*=1 animal, *n*=1 marine), and all encoding a single ColRNAI_rep_cluster_1987 replicon and no known relaxases, AMR or virulence genes. However, C1_S438A remained a diverse group of 62 plasmids (*n*=56 human, *n*=2 animal, *n*=4 marine) ranging in length from 87.1 to 282.6 kbp: all harboured three replicon markers, one of which was IncFII (followed in declining frequency by IncFIB, IncFIA, IncQ1, rep_cluster2183 and/or rep_cluster_1418). C1_S440 was also divided into smaller clusters. C1_S440A contained 95 plasmids spanning all three niches, all of which were <11 kbp and lacked a relaxase, and most of which were predicted to be non-mobilizable with a ColRNAI_rep_cluster_1987 replicon marker. While the plasmids assigned to clusters C1_S440B-F were considerably larger (32–424 kbp) and represented all three niches, AMR genes (*bla*_CTX-M-15_, *bla*_KPC-2_, genes encoding resistance to aminoglycosides, etc.) were only present in plasmids from human sources.

### The Norwegian KpSC plasmids are representative in a global context

**Fig. 6.**
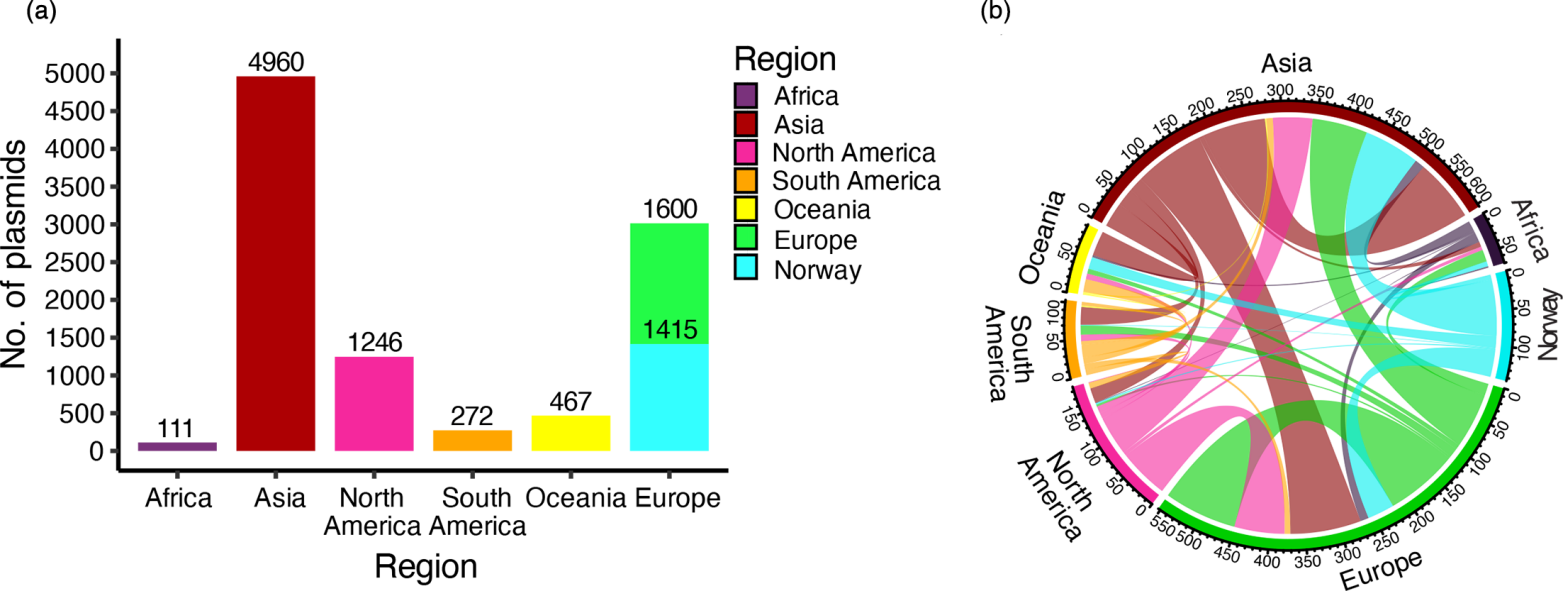
Distribution of global KpSC plasmids. (**a**) Bar plots indicate the total number of plasmids included in global clustering analysis from each world region. (**b**) Chord diagram of plasmids assigned to the same clusters and found in multiple geographic locations. See Fig. S11 for alternative world map views.

To contextualize the Norwegian KpSC plasmidome within a broader genomic landscape, we compared our collection to 8,656 circularized plasmids from publicly available KpSC genomes downloaded from the NCBI RefSeq (see details and inclusion criteria in Methods), collected from 65 countries in six world regions between 2015 and 2024 (Fig. 6a, Table S2). Nearly half of the plasmids were from isolates collected in Asia (*n*=4,960), followed by Europe (*n*=3,015, including plasmids from this study), North America (*n*=1,246), Oceania (*n*=467), South America (*n*=272) and Africa (*n*=111), resulting in a total of *n*=10,071 plasmids in the global dataset (Fig. 6a). To identify overlap between our collection and the global dataset, the European region was further divided into: ‘Europe’ (*n*=1,600, including six Norwegian plasmids not from this study) and ‘Norway’ (plasmids in this study, *n*=1,415).

Clustering with Pling resulted in 90 communities, 614 clusters, 1,544 singletons (15.3%) and 362 hub plasmids (3.6%) (Fig. S10). Interestingly, plasmids from our Norwegian collection were broadly dispersed throughout the global network: they were present in 66.1% of multi-region communities (*n*=37/56) and 63.2% of multi-region clusters (*n*=172/272). This distribution indicates broad overlap between plasmids in this dataset and the global KpSC plasmid landscape (Fig. 6b). In total, 9 communities (10.0%), 42 clusters (6.8%) and 396 singletons (25.6%) consisted exclusively of plasmids from this study. As with the national dataset, the global network was dominated by a single large community that accounted for the vast majority of plasmids in the dataset (92.0%, *n*=9,264/10,071). Most communities (62.2%, *n*=56/90) included plasmids from more than one world region. Excluding hub plasmids, 44.3% of clusters (*n*=272/614) contained plasmids from multiple regions (Fig. 6b), including eight clusters that encompassed all six regions and together represented 22.4% of all plasmids (*n*=2,255/10,071).

Using STs as the available lineage marker for this collection, we assessed if some lineages harboured a wider range of plasmid types than others and if some plasmid clusters showed broader host ranges, following the same framework applied to the Norwegian plasmid clusters. There were 675 STs in the global dataset, 440 of which were represented by a single isolate (65.2%). Each ST contained between 1 and 280 (median=3, mean=6.37) unique plasmid types (including clusters and singletons).

Plasmid clusters (excluding hubs) were distributed amongst 1–246 STs (median=2, mean=4.35). Many clusters (38.3%, *n*=235/614) were restricted to a single ST, while 379 plasmid clusters were distributed across multiple STs. There were 58 clusters with plasmids present in ≥10 STs, nearly half of which (41.4%, *n*=24/58) were clusters primarily composed of small (<25 kbp) cryptic plasmids (mean<24 kbp, median<11 kbp). Among these predominantly cryptic clusters, 12 spanned more than one global region and contained at least one plasmid encoding AMR. Plasmids belonging to these 12 clusters were present in a total of 1,515 isolates.

Excluding hub plasmids, a total of 328 clusters and 567 singletons contained plasmids harbouring either AMR and/or virulence genes. Of these, 282 clusters and 461 singletons contained plasmids encoding AMR, 38 clusters and 59 singletons contained virulence plasmids and 32 clusters and 47 singletons contained plasmids encoding both AMR genes and virulence factors. Among hub plasmids (*n*=362), 61.6% (*n*=223) carried AMR genes, 0.8% (*n*=3) encoded virulence factors and 8.6% (*n*=31) encoded both.

Of the 282 clusters containing AMR plasmids, 42.6% (*n*=120) spanned more than one global region, 34 of which also contained AMR plasmids from our dataset. However, many plasmids from this dataset that did not encode AMR genes were also found in global clusters containing AMR plasmids from other global regions, demonstrating that plasmid backbones lacking AMR genes locally are associated with AMR plasmids globally. Most of the 38 clusters containing virulence-only plasmids spanned multiple regions (52.6%, *n*=20), and 14 of those also contained virulence plasmids from this dataset, with each virulence locus represented in ≥1 cluster. Of the 31 clusters containing plasmids that encoded both AMR and virulence, 54.8% (*n*=17) spanned two to six global regions (two regions, *n*=5 clusters; three regions, *n*=5; four regions, *n*=1; five regions, *n*=3; six regions, *n*=3), of which only one cluster contained plasmids from our dataset.

## Discussion

This study provides a comprehensive analysis of the plasmid diversity and associated AMR and virulence factors in KpSC isolates across three ecological niches in Norway. Our findings demonstrate the extensive diversity and adaptability of KpSC plasmids, underlining their role in AMR and virulence transmission within and across sectors. The combination of high structural variability, niche-specific selection and widespread cross-niche clustering observed in this study allows a broader interpretation of how the KpSC plasmidome evolves and persists across environments.

The variation in plasmid load across niches, with human and marine isolates harbouring significantly greater plasmid loads than animal isolates, suggests that selective pressures in human-associated and coastal marine environments may favour retention of multiple plasmids or higher plasmid copy numbers, potentially reflecting differences in antibiotic exposure or other environmental stressors. Additionally, as KpSC marine isolates were predominantly detected near coastlines rather than in open water [18], it is likely that isolates and plasmids from human and marine sources originated from similar backgrounds as a result of wastewater and/or terrestrial runoff, or via human consumption of seafood [43,44]. Though not a direct indication of HGT itself, this pattern implies plasmid persistence in new environments. While plasmid diversity was widespread across KpSC species, ecological niche appeared to play a more significant role in shaping plasmid contents.

Niche-specific patterns were particularly evident in the distribution of AMR and virulence plasmids. The human niche, as expected, had a significantly higher prevalence of AMR plasmids, likely reflecting the impact of human antibiotic use [3,5,45] relative to the lower use of antibiotics in the agricultural sector in our setting [49,50] and aligning with patterns reported in international genomic One Health studies [5,46, 47]. In contrast, the animal niche was enriched for virulence-encoding plasmids, specifically those carrying *iuc*3 and *iuc*5. The *iuc*3 locus has previously been linked to pig-associated lineages [24,41], while *iuc*5 was associated with a clonal expansion in turkeys [25]. Interestingly, these were predominantly found in non-HV clones [1], highlighting the potential role of animal reservoirs in the evolution of virulent KpSC strains that may cross into human populations. Conversely, *iuc*1 and *iuc*2 plasmids were found exclusively in the human niche and were associated with globally recognized HV clones and even some MDR lineages [1]. These contrasting distributions suggest ecological filtering, where selective pressures – such as antibiotic exposure in human settings or host–pathogen interactions in livestock – favour maintenance of distinct plasmid types. The distribution of clinically relevant plasmids across both high-risk MDR clones and other SLs demonstrates the capacity of KpSC to acquire and retain plasmids across diverse clonal backgrounds, suggesting that acquisition of such plasmids is not confined to high-risk clonal groups.

Clinically relevant genes were frequently co-carried with well-characterized replicon markers across all niches. In particular, plasmids encoding *bla*_CTX-M-3_, *bla*_CTX-M-14_ and *bla*_CTX-M-15_ were consistently associated with IncFIB and IncFII, while siderophore loci (*iro*, *iuc*, excluding *iuc*2A) and capsule regulators (*rmpA*, *rmpA2*) were linked to IncFIB(K) and IncFIB. Although most AMR-encoding plasmids were predicted to be conjugative or mobilizable, several *iuc*1-carrying plasmids (in both the Norwegian and global plasmid collections) lacked conjugation machinery and were predicted to be non-mobilizable, consistent with previous findings by Lam *et al.* [10], suggesting alternative dissemination routes such as clonal expansion or mobilization by co-resident plasmids. The association of MOBP and MOBF relaxases with ESBL- and siderophore-encoding plasmids further supports their role in mobilizing clinically relevant plasmids across ecological and genomic contexts [14], illustrating the modular nature of plasmid gene content, where combinations of replicon and relaxase types determine both stability and mobility.

The increased TE burden on AMR plasmids compared to their non-AMR counterparts suggests that TEs play a disproportionate role in AMR gene acquisition and structural rearrangement and are not merely passive genomic material but active contributors to plasmid evolution, consistent with the link between IS abundance and AMR carriage reported in previous studies [13,14, 51]. The enrichment of multiple IS elements on several 120–250 kbp plasmids encoding both AMR and virulence factors also aligns with the recognized role of TEs in facilitating convergence events [14,51]. Together, these findings support current models in which replicon type, MOB machinery and TE content shape both the composition and dissemination potential of clinically important plasmids, with structural plasticity itself serving as an adaptive trait that enables rapid gene turnover under shifting selective pressures.

A significant proportion (37.0%) of the Norwegian collection consisted of singletons, reflecting a highly dynamic plasmidome. Additionally, many plasmids (42.9%) were assigned to multi-niche clusters or shared considerable sequence content with global counterparts, indicating that many plasmids are capable of persisting and spreading across broad ecological and geographical ranges. The abundance of unique plasmids and the absence of conserved core genes in several clusters imply rapid structural diversification, consistent with ongoing recombination and modular gain or loss of accessory genes, evolutionary processes that generate new plasmid variants even within single ecological settings. In some clusters, this diversification occurred despite narrow plasmid size ranges, suggesting frequent gene turnover and rearrangement even within structurally conserved backbones.

Despite Norway’s relatively low antimicrobial usage across multiple sectors [49,52], Norwegian plasmids showed substantial overlap with the global KpSC plasmidome, with plasmids from this collection present in over half of the global Pling clusters. While many of the Norwegian plasmids lacked AMR genes, they frequently clustered with globally distributed AMR plasmids, suggesting that gene-acquisition-ready plasmids are already circulating in low-AMR settings. These patterns underscore the global interconnectedness of plasmid evolution and highlight the importance of One Health-informed plasmid backbone surveillance.

Whereas our previous study [6] showed limited strain-sharing between niches (*n*=9 strain-sharing events among *n*=3,255 isolates), the present analysis reveals greater plasmid overlap (*n*=47 Pling clusters) across the same reservoirs, consistent with the recognized ability of plasmids to traverse ecological boundaries more readily than their host strains [3,4, 13, 47]. Accordingly, plasmid-sharing was considerably more common across niches than strain-sharing, reflecting their capacity for horizontal transfer and persistence across diverse genomic backgrounds even when strain-level transmission is comparatively rare.

Plasmid distribution across SLs also highlighted distinct strategies of plasmid persistence. Within our dataset, the plasmid clusters that spanned the greatest number of SLs were predominantly very small (<15 kbp) plasmids, most of which lacked conjugation machinery yet were often mobilizable. Their broad host-range distribution suggests that small plasmids with low fitness cost can persist across diverse lineages, likely via mobilization by co-resident conjugative plasmids or through stable vertical inheritance. In contrast, only three clusters of medium- to large-sized plasmids displayed similarly broad SL ranges, representing successful backbone types in unrelated lineages. These included a cluster of large (~155 kbp), conjugative, virulence-associated (*iuc*3) plasmids and two clusters of medium-sized (60–80 kbp) plasmids with contrasting predicted mobility profiles – one in which most plasmids carried known relaxases and were predicted mobilizable, and another in which no known relaxases were detected and all plasmids were predicted non-mobilizable. Although small, cryptic plasmids in our Norwegian dataset did not encode AMR or virulence determinants, globally these plasmid backbones were widespread: clusters of predominantly small (<25 kbp) plasmids were present in 1,515 isolates across two to six world regions. Only a small minority of plasmids within these clusters carried AMR genes, yet their broad distribution demonstrates that these small backbones form established dissemination networks. This suggests that rare AMR-acquisition events in cryptic plasmids could propagate widely once established, consistent with concerns raised for small *Klebsiella* plasmids [53].

There are several strengths and limitations in this study. While this study includes a large, hybrid-assembled dataset of KpSC isolates spanning three ecological niches and two decades across Norway, the collection was skewed towards human isolates due to availability, and plasmid-rich isolates were prioritized for long-read sequencing. However, ~20% of the collected samples from each niche were long-read sequenced, ensuring proportional representation. The reliance on isolates collected primarily from human sources likely influenced the observed distribution of plasmid types and associated genetic elements. Moreover, while our global comparison included high-quality complete genomes, it remains constrained by public database biases, particularly overrepresentation of clinical isolates, imbalanced representation of world regions and underreporting of ecological metadata. Importantly, while multi-niche and multi-region plasmid clusters were identified, we did not perform epidemiological analyses to investigate transmission routes across regions or niches.

In both the national and global analyses, our clustering approach identified structural relatedness but did not provide evidence of direct plasmid transfer, nor did it resolve directionality or timing of dissemination events. These constraints should be addressed in future work using more extensive sampling from underrepresented hosts, environments and geographical locations, along with longitudinal monitoring to track plasmid evolution. Further investigations into the functional impact of plasmid diversity – particularly of small, cryptic plasmids and phage-plasmids – are needed, as these may play underappreciated roles in HGT. Experimental validation of plasmid fitness effects in different contexts would also illuminate the mechanisms underlying their persistence and spread.

Nonetheless, this study represents one of the largest and most comprehensively analysed plasmid datasets from a national One Health perspective. The use of hybrid assembly and plasmid clustering allowed us to resolve full plasmid structures, enabling comparative analyses at a high resolution. The high degree of overlap between our plasmid collection and global plasmid clusters highlights the utility of such national collections in global AMR surveillance efforts and underscores the global relevance of plasmid populations from low-AMR settings. Our findings indicate that plasmid backbones form a persistent, globally connected reservoir for adaptive genes, emphasizing the need for surveillance beyond strain typing.

Collectively, our results portray the KpSC plasmidome as an evolving network of modular genetic elements shaped by both HGT and ecological selection. Structural diversity, high TE activity and recurrent multi-niche clustering indicate that plasmids undergo continual recombination and adaptation. Rather than evolving solely through lineage inheritance, plasmids appear to persist as dynamic mosaics that bridge ecological and taxonomic boundaries. Furthermore, the Norwegian plasmid collection was globally representative, demonstrating that similar plasmid backbones capable of acquiring and transmitting clinically significant traits are already circulating across human, animal and marine niches, as well as in both low- and high-AMR regions. These findings suggest long-term stability of these mobile platforms that persist and disseminate independently of local antibiotic pressure and emphasize the importance of continued plasmid-level surveillance and integrated One Health strategies to prevent the spread of AMR and virulence traits.

## Supplementary material

10.1099/mgen.0.001629 Uncited Supplementary Material 1.

10.1099/mgen.0.001629 Uncited Supplementary Material 2.

## Abbreviations

AMR: antimicrobial resistance
DCJ-indel: double cut and join insertion–deletion
ESBLs: extended-spectrum *β*-lactamases
HGT: horizontal gene transfer
HMR: heavy metal resistance
HV: hypervirulence
ISs: insertion sequences
KpSC: *Klebsiella pneumoniae* species complex
MDR: multidrug resistance
ONT: Oxford nanopore technologies
SLs: sublineages
STs: sequence types
TEs: transposable elements
