# Supplementary material for: A One Health study of Klebsiella pneumoniae species complex plasmids shows a highly diverse and ecologically adaptable plasmidome
Source: Microb Genom. 2026 Feb 19;12(2):001629. doi: 10.1099/mgen.0.001629 (PMC12919850; doi:10.1099/mgen.0.001629)
Supplement: Uncited Supplementary Material 1. [file mgen-12-01629-s001.pdf]

Supplementary Material

A One Health study of *Klebsiella pneumoniae* species complex plasmids shows a highly diverse and ecologically adaptable plasmidome

Mia A Winkler [0009-0003-4838-6838] [1, 2, a], Marit A K Hetland [0000-0003-4247-8304] [2, 3], Håkon Pedersen Kaspersen [0000-0002-9559-1303] [4], Ragna-Johanne Bakksjø [0000-0002-2921-7866] [2], Eva Bernhoff [0000-0001-6715-757X] [2], Aasmund Fostervold [0000-0001-5353-2084] [2, 5], Jane Hawkey [0000-0001-9661-5293] [6, 7], Bjørn-Tore Lunestad [0000-0002-9139-8346] [8, 9], Nachiket P Marathe [0000-0003-2955-3402] [8], Niclas Raffelsberger [0000-0002-9463-8915] [1, 10], Ørjan Samuelsen [0000-0002-5525-2614] [11], Marianne Sunde [0000-0002-2326-4338] [12], Arnfinn Sundsfjord [0000-0002-3728-2270] [1, 11], Margaret M C Lam [0000-0001-9590-6849] [6, 7, b] and Iren H Löhr [0000-0002-8847-0044] [2, 5, b]

[1] Department of Medical Biology, Faculty of Health Sciences, UiT The Arctic University of Norway, Tromsø, Norway

[2] Department of Medical Microbiology, Stavanger University Hospital, Stavanger, Norway

[3] Department of Biological Sciences, Faculty of Science and Technology, University of Bergen, Bergen, Norway

[4] Research Section Food Safety and Animal Health, Department of Animal Health and Food Safety, Norwegian Veterinary Institute, Oslo, Norway

[5] Department of Clinical Science, Faculty of Medicine, University of Bergen, Bergen, Norway

[6] Department of Infectious Diseases, Central Clinical School, Monash University, Melbourne, Australia

[7] Centre to Impact AMR, Monash University, Melbourne, Australia

[8] Institute of Marine Research, Bergen, Norway

[9] Department of Biological Sciences, University of Bergen, Bergen, Norway

[10] Department of Microbiology and Infection Control, University Hospital of North Norway, Tromsø, Norway

[11] Norwegian Centre for Detection of Antimicrobial Resistance, Department of Microbiology and Infection Control, University Hospital of North Norway, Tromsø, Norway

[12] Section for Bacteriology, Department for Analysis and Diagnostics, Norwegian Veterinary Institute, Ås, Norway

[a] Corresponding author: Mia A. Winkler, mia.winkler@uit.no; mia.angelique.winkler@sus.no

[b] These authors contributed equally

Keywords: *Klebsiella pneumoniae*; plasmids; antimicrobial resistance; One Health

Contents

Supplementary Methods

See pages 3-8 of this document for methods supplementary to the main text

Supplementary Figures

Fig. S1. Plasmid distribution by species.
Fig. S2. Overlap of clinically relevant features across whole genomes.
Fig. S3. Co-occurrence of clinically relevant features by plasmid.
Fig. S4. Distribution of plasmid-encoded features across plasmid lengths.
Fig. S5. Overlap of clinically relevant features with replicon and MOB types.
Fig. S6. Prevalence of transposable elements (TEs) across plasmid categories.
Fig. S7. Full Pling network graph (n=1415 plasmids) including all communities, clusters, singletons, and hub plasmids.
Fig. S8. Comparison of strain- and plasmid-sharing within and between niches.
Fig. S9. Frequency of Pling cluster and SL overlap.
Fig. S10. Pling network graph of global and local plasmids.
Fig. S11. Geographical overlap of plasmid clusters.

Supplementary Tables

Please see separate Excel file for the Supplementary Tables:

Table S1: Norwegian isolate metadata and genotyping results
Table S2: Global dataset accessions and genotyping

Supplementary Methods

Sample selection for long-read sequencing

The isolates selected for long-read sequencing were part of a larger dataset (n=3255) that had been previously short-read sequenced in several published studies (1–10). To generate a representative subset of *Klebsiella pneumoniae* species complex (KpSC) isolates for hybrid genome assembly, we applied a multi-step selection process from the initial collection of 3,255 isolates. Our aims were to ensure a representative selection of the dataset, capture clinically relevant antimicrobial resistance and/or virulence genes, and broad representation of the plasmid diversity, while optimizing sequencing efficiency.

The initial selection of 359 genomes prioritized representativeness of the overall collection after analysis with Bakta v1.8.1 with database v5.0 (11), Panaroo v1.3.3 (12), and a long-read selection pipeline (https://gitlab.com/sirarredondo/long_read_selection), as well as ensuring each sublineage (SL) that overlapped the three ecological niches (human, animal, and marine) was represented at least once in order to maximise diversity within the resulting dataset (10).

We reviewed the draft assemblies and raw reads of the remaining 2896/3255 isolates to select an additional 192 isolates with the aim of maximizing plasmid diversity and capturing clinically relevant features. Raw reads were assessed using SRST2 v0.2.0 (13) with the PlasmidFinder plasmidfinder-db v2023-01-18 (14) and MOB-typer v3.1.5 (15) databases, and draft assemblies were assessed using Abricate v1.0.1 (https://github.com/tseemann/abricate) with the same databases. Isolates were prioritized if they carried a diverse range of replicon markers and/or MOB types. To ensure comprehensive plasmid representation, at least one isolate was selected to represent each replicon and relaxase type, with additional selections for markers appearing across multiple plasmids. Any isolate which contained the only instance of a given plasmid marker in either the raw reads or draft assembly was automatically selected for long-read sequencing. Niche and phylogenetic diversity were also considered in the selection process. Isolates were chosen to represent all three ecological niches, with additional emphasis on traits spanning multiple niches when possible. Representation of multiple KpSC members was also incorporated to ensure broad genomic representation across the dataset. We included isolates where plasmid markers were present in raw reads but absent from draft assemblies, ensuring these sequences were not lost due to assembly artefacts. This resulted in a total of

578 hybrid-assembled isolates, of which 514 contained a total of 1427 plasmids (n=1415 complete circularised, n=1 complete linear, n=11 unclosed plasmid sequences).

Heavy metal resistance genes

To identify heavy metal resistance (HMR) genes, we searched the annotated plasmid assemblies for genes listed in the Antibacterial Biocide and Metal Resistance Genes (BacMet) database (http://bacmet.biomedicine.gu.se/index.html) (16). Based on literature searches, we used the following operons to determine presence or absence of heavy metal resistance: Arsenic resistance was determined by the presence of *arsAD*, *arsBCR*, or *arsABCDR* (17–19). Resistance to chromium was defined by the presence of *chrA* or *chrAB* (17,20,21). Mercury resistance was determined by the presence of at least *merAPR* and either *merC*, *merF*, or *merT* (17,22,23). Copper resistance was defined as *pcoABCDRS* (17,24). The *ncrABC* operon was used to define nickel resistance (25). Silver resistance was defined as the presence of at minimum *silABCERS* (17). The *terBCDE* operon was used to determine tellurite resistance (26). The presence of *rcnAR* defined resistance to cobalt and nickel (27). The *zitB* gene indicated resistance to zinc (28).

Plasmid clustering with Pling

The closed plasmids were clustered with Pling (29), which operates in three main stages: 1) construction of a containment network based on the proportion of sequence shared between two plasmids (i.e. their containment distance), 2) calculation of structural distances using the double-cut and join indel (DCJ-indel) model, and 3) refinement of the plasmid network by identifying hub plasmids.

To identify plasmid pairs that share sufficient sequence for meaningful structural comparison, Pling calculates a containment distance between each pair, which is defined as the proportion of the smaller plasmid not aligned to the larger of the two. By default, plasmids with a containment distance ≤0.5 are assigned to the same community and retained for further comparison.

Plasmid pairs within the containment threshold are then integerised, wherein each sequence is converted to an ordered list of integers representing synteny blocks; shared blocks are assigned the same integer across plasmids. By default, unaligned regions ≥200 bp are treated as indels and receive unique identifiers.

Using the integerised representations, Pling computes the DCJ-indel distance between plasmids, which reflects the minimal number of structural operations (i.e. rearrangements, including inversions, translocations, insertions, and deletions) needed to convert one plasmid structure into another. By default, plasmids with ≤4 rearrangements are assigned to the same subcommunity, which represents the currently evolving unit of those plasmids. These subcommunities were referred to as plasmid clusters.

Hub plasmids are defined as highly connected nodes that bridge otherwise unconnected groups. By default, these are identified as plasmids with node degree >10 and the neighbouring node's edge density <0.2, where edge density is the proportion of observed edges among neighbours relative to the maximum possible. Pling iterates over the DCJ-indel network, first removing hub plasmids in the original network, then identifying and removing hubs induced by removal of the first set, etc., until no more hubs remain in the network. Hubs often represent plasmids dominated by transposable elements (TEs), which can inappropriately inflate network connectivity.

References

1. Håkonsholm F, Hetland MAK, Svanevik CS, Sundsfjord A, Lunestad BT, Marathe NP. Antibiotic Sensitivity Screening of Klebsiella spp. and Raoultella spp. Isolated from Marine Bivalve Molluscs Reveal Presence of CTX-M-Producing K. pneumoniae. Microorganisms. 2020 Dec;8(12):1909.

2. Franklin-Alming FV, Kaspersen H, Hetland MAK, Bakksjø RJ, Nesse LL, Leangapichart T, et al. Exploring Klebsiella pneumoniae in Healthy Poultry Reveals High Genetic Diversity, Good Biofilm-Forming Abilities and Higher Prevalence in Turkeys Than Broilers. Front Microbiol [Internet]. 2021 Sep 7 [cited 2025 Jul 1];12. Available from: https://www.frontiersin.org/journals/microbiology/articles/10.3389/fmicb.2021.725414/full

3. Raffelsberger N, Hetland MAK, Svendsen K, Småbrekke L, Löhr L Iren Høyland, Andreassen LLE, et al. Gastrointestinal carriage of Klebsiella pneumoniae in a general adult population: a cross-sectional study of risk factors and bacterial genomic diversity. Gut Microbes. 2021 Jan 1;13(1):1939599.

4. Fostervold A, Hetland MAK, Bakksjø R, Bernhoff E, Holt KE, Samuelsen Ø, et al. A nationwide genomic study of clinical Klebsiella pneumoniae in Norway 2001–15: introduction and spread of ESBLs facilitated by clonal groups CG15 and CG307. Journal of Antimicrobial Chemotherapy. 2022 Mar 2;77(3):665–74.

5. Håkonsholm F, Hetland MAK, Svanevik CS, Lunestad BT, Löhr IH, Marathe NP. Insights into the genetic diversity, antibiotic resistance and pathogenic potential of *Klebsiella pneumoniae* from the Norwegian marine environment using whole-genome analysis. International Journal of Hygiene and Environmental Health. 2022 May 1;242:113967.

6. Håkonsholm F, Hetland MAK, Löhr IH, Lunestad BT, Marathe NP. Co-localization of clinically relevant antibiotic- and heavy metal resistance genes on plasmids in Klebsiella pneumoniae from marine bivalves. MicrobiologyOpen. 2023;12(4):e1368.

7. Kaspersen H, Franklin-Alming FV, Hetland MAK, Bernhoff E, Löhr IH, Jiwakanon J, et al. Highly conserved composite transposon harbouring aerobactin iuc3 in Klebsiella pneumoniae from pigs. Microbial Genomics. 2023;9(2):000960.

8. Kaspersen H, Urdahl AM, Franklin-Alming FV, Ilag HK, Hetland MAK, Bernhoff E, et al. Population dynamics and characteristics of Klebsiella pneumoniae from healthy poultry in Norway. Front Microbiol [Internet]. 2023 May 18 [cited 2024 Sep 10];14. Available from: https://www.frontiersin.org/journals/microbiology/articles/10.3389/fmicb.2023.1193274/full

9. Fostervold A, Raffelsberger N, Hetland MAK, Bakksjø R, Bernhoff E, Samuelsen Ø, et al. Risk of death in Klebsiella pneumoniae bloodstream infections is associated with specific phylogenetic lineages. Journal of Infection [Internet]. 2024 May 1 [cited 2024 Sep 10];88(5). Available from: https://www.journalofinfection.com/article/S0163-4453(24)00089-6/fulltext

10. Hetland MAK, Winkler MA, Kaspersen HP, Håkonsholm F, Bakksjø RJ, Bernhoff E, et al. A genome-wide One Health study of Klebsiella pneumoniae in Norway reveals overlapping populations but few recent transmission events across reservoirs. Genome Medicine. 2025 Apr 28;17(1):42.

11. Schwengers O, Jelonek L, Dieckmann MA, Beyvers S, Blom J, Goesmann A. Bakta:

rapid and standardized annotation of bacterial genomes via alignment-free sequence identification. Microbial Genomics. 2021;7(11):000685.

12. Tonkin-Hill G, MacAlasdair N, Ruis C, Weimann A, Horesh G, Lees JA, et al. Producing polished prokaryotic pangenomes with the Panaroo pipeline. Genome Biology. 2020 Jul 22;21(1):180.

13. Inouye M, Dashnow H, Raven LA, Schultz MB, Pope BJ, Tomita T, et al. SRST2: Rapid genomic surveillance for public health and hospital microbiology labs. Genome Medicine. 2014 Nov 20;6(11):90.

14. Carattoli A, Zankari E, García-Fernández A, Voldby Larsen M, Lund O, Villa L, et al. In Silico Detection and Typing of Plasmids using PlasmidFinder and Plasmid Multilocus Sequence Typing. Antimicrob Agents Chemother. 2014 Jul;58(7):3895–903.

15. Robertson J, Nash JHE. MOB-suite: software tools for clustering, reconstruction and typing of plasmids from draft assemblies. Microbial Genomics. 2018;4(8):e000206.

16. Pal C, Bengtsson-Palme J, Rensing C, Kristiansson E, Larsson DGJ. BacMet: antibacterial biocide and metal resistance genes database. Nucleic Acids Research. 2014 Jan 1;42(D1):D737–43.

17. Silver S, Phung LT. A bacterial view of the periodic table: genes and proteins for toxic inorganic ions. Journal of Industrial Microbiology and Biotechnology. 2005 Dec 1;32(11–12):587–605.

18. Mukhopadhyay R, Rosen BP, Phung LT, Silver S. Microbial arsenic: from geocycles to genes and enzymes. FEMS Microbiology Reviews. 2002 Aug 1;26(3):311–25.

19. González Henao S, Ghneim-Herrera T. Heavy Metals in Soils and the Remediation Potential of Bacteria Associated With the Plant Microbiome. Front Environ Sci [Internet]. 2021 Apr 12 [cited 2025 Jul 1];9. Available from: https://www.frontiersin.org/journals/environmental-science/articles/10.3389/fenvs.2021.604216/full

20. Pimentel BE, Moreno-Sánchez R, Cervantes C. Efflux of chromate by Pseudomonas aeruginosa cells expressing the ChrA protein. FEMS Microbiology Letters. 2002 Jul 1;212(2):249–54.

21. Cervantes C, Campos-García J, Devars S, Gutiérrez-Corona F, Loza-Tavera H, Torres-Guzmán JC, et al. Interactions of chromium with microorganisms and plants. FEMS Microbiology Reviews. 2001 May 1;25(3):335–47.

22. Barkay T, Miller SM, Summers AO. Bacterial mercury resistance from atoms to ecosystems. FEMS Microbiology Reviews. 2003 Jun 1;27(2–3):355–84.

23. Boyd E, Barkay T. The Mercury Resistance Operon: From an Origin in a Geothermal Environment to an Efficient Detoxification Machine. Frontiers in Microbiology [Internet]. 2012 [cited 2024 Jan 20];3. Available from: https://www.frontiersin.org/articles/10.3389/fmicb.2012.00349

24. Rensing C, Grass G. Escherichia coli mechanisms of copper homeostasis in a changing environment. FEMS Microbiology Reviews. 2003 Jun 1;27(2–3):197–213.

25. Hufnagel DA, Choby JE, Hao S, Johnson AF, Burd EM, Langelier C, et al. Antibiotic-

Selected Gene Amplification Heightens Metal Resistance. mBio. 2021 Jan 19;12(1):10.1128/mbio.02994-20.

26. Chasteen TG, Fuentes DE, Tantaleán JC, Vásquez CC. Tellurite: history, oxidative stress, and molecular mechanisms of resistance. FEMS Microbiology Reviews. 2009 Jul 1;33(4):820–32.

27. Rodrigue A, Effantin G, Mandrand-Berthelot MA. Identification of rcnA (yohM), a nickel and cobalt resistance gene in Escherichia coli. Journal of Bacteriology. 2005 Apr;187(8):2912–6.

28. Grass G, Fan B, Rosen BP, Franke S, Nies DH, Rensing C. ZitB (YbgR), a Member of the Cation Diffusion Facilitator Family, Is an Additional Zinc Transporter in Escherichia coli. J Bacteriol. 2001 Aug;183(15):4664–7.

29. Frolova D, Lima L, Roberts LW, Bohnenkämper L, Wittler R, Stoye J, et al. Applying rearrangement distances to enable plasmid epidemiology with pling. Microbial Genomics. 2024;10(10):001300.

Supplementary Figures

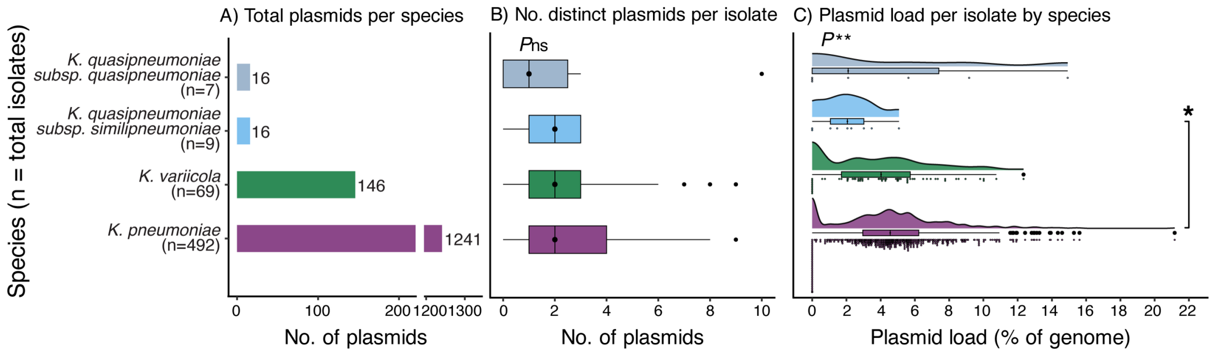

Fig. S1. Plasmid distribution by species. A) Total number of plasmids per species. **B)** Distribution of the number of distinct plasmid sequences per isolate in each KpSC species, excluding *Klebsiella quasivariicola* (n=1); the black vertical line indicates median value. **C)** Distribution of estimated plasmid load per isolate within each species, excluding *K. quasivariicola*. Statistical comparisons were performed using Kruskal-Wallis (overall) and Mann-Whitney (pairwise) tests. Significance is denoted as follows: *P<0.05, ns P≥0.05.

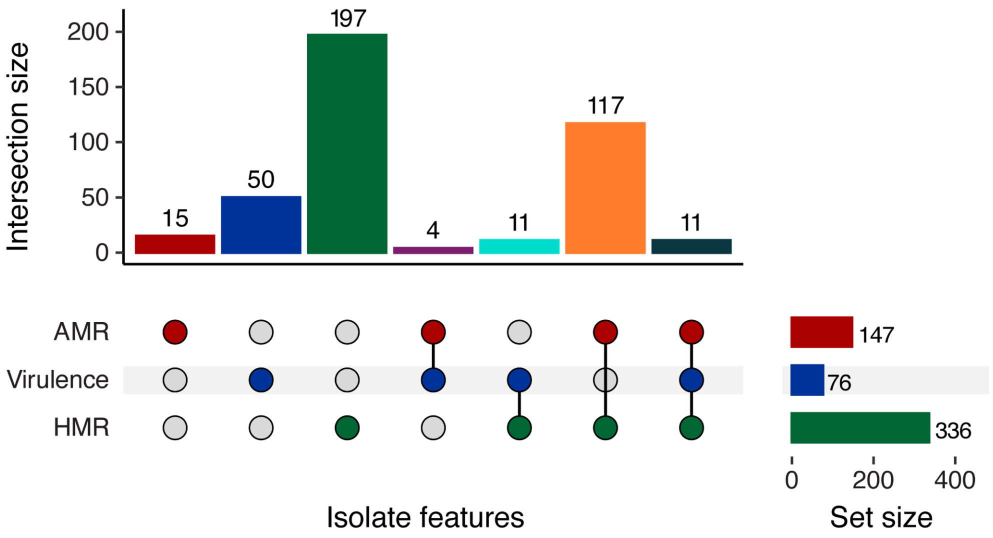

Fig. S2. Overlap of clinically relevant features across whole genomes. Upset plot showing the overlap of plasmid-encoded clinically relevant genetic features across n=405/514 plasmid-harbouring isolates. Antimicrobial resistance (AMR) in red, virulence factors in blue, and heavy metal resistance (HMR) in green.

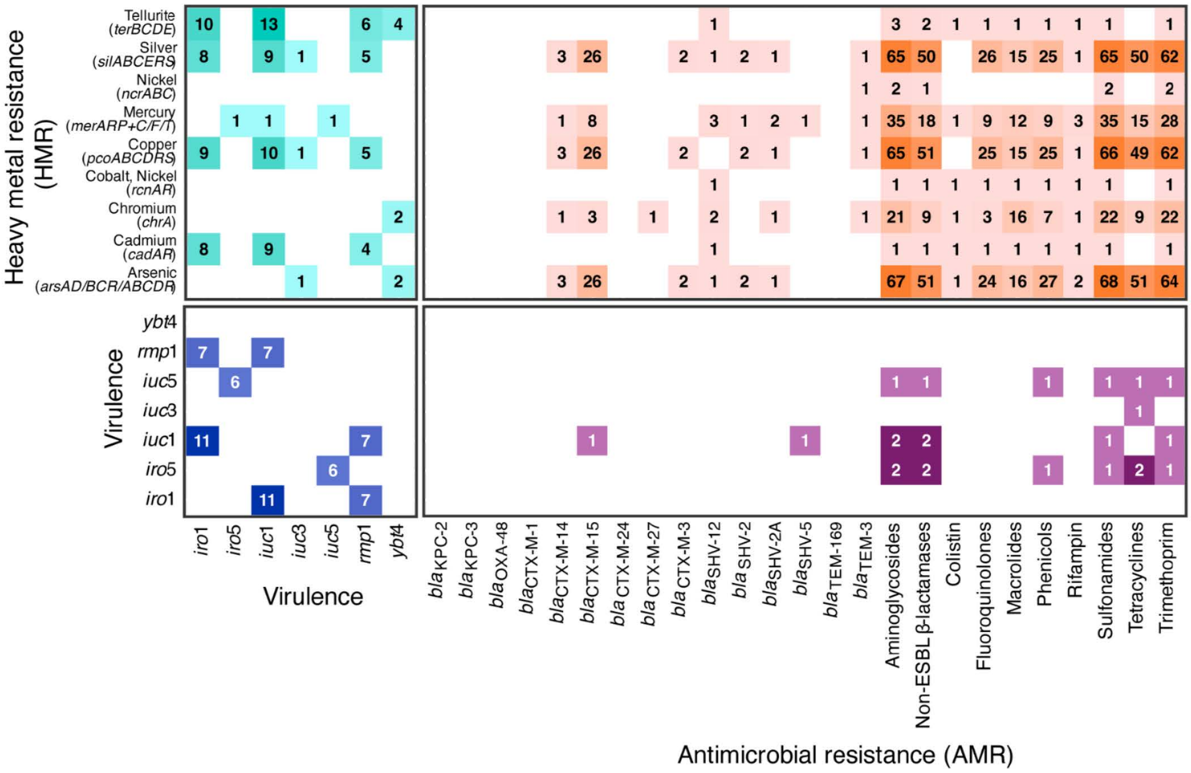

Fig. S3. Co-occurrence of clinically relevant features by plasmid. Frequency of clinically relevant traits residing on the same plasmid: heavy metal resistance (HMR) and virulence (green), HMR and AMR class or AMR genes (for ESBLs and carbapenemases) (orange), virulence and AMR (purple), and overlapping virulence factors (blue). Not shown: virulence factors or HMR operons that did not overlap with either of the other two categories.

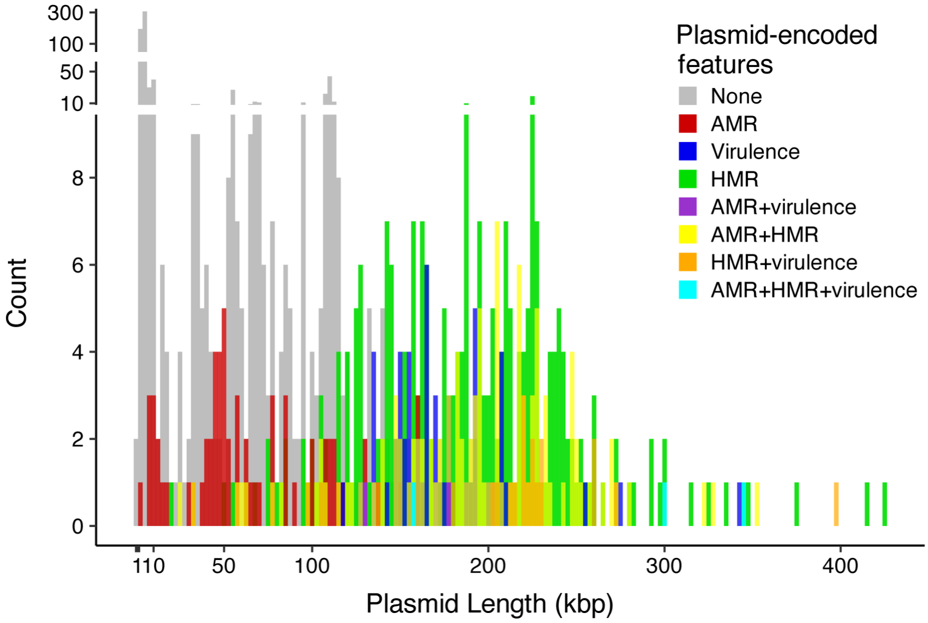

Fig. S4. Distribution of plasmid-encoded features across plasmid lengths. Each bar shows the number of plasmids in a given length range (x-axis) carrying genes for AMR (red), virulence (blue), AMR and virulence (purple), heavy metal resistance (HMR) (green), AMR and HMR (yellow), virulence and HMR (orange), or all three (light blue).

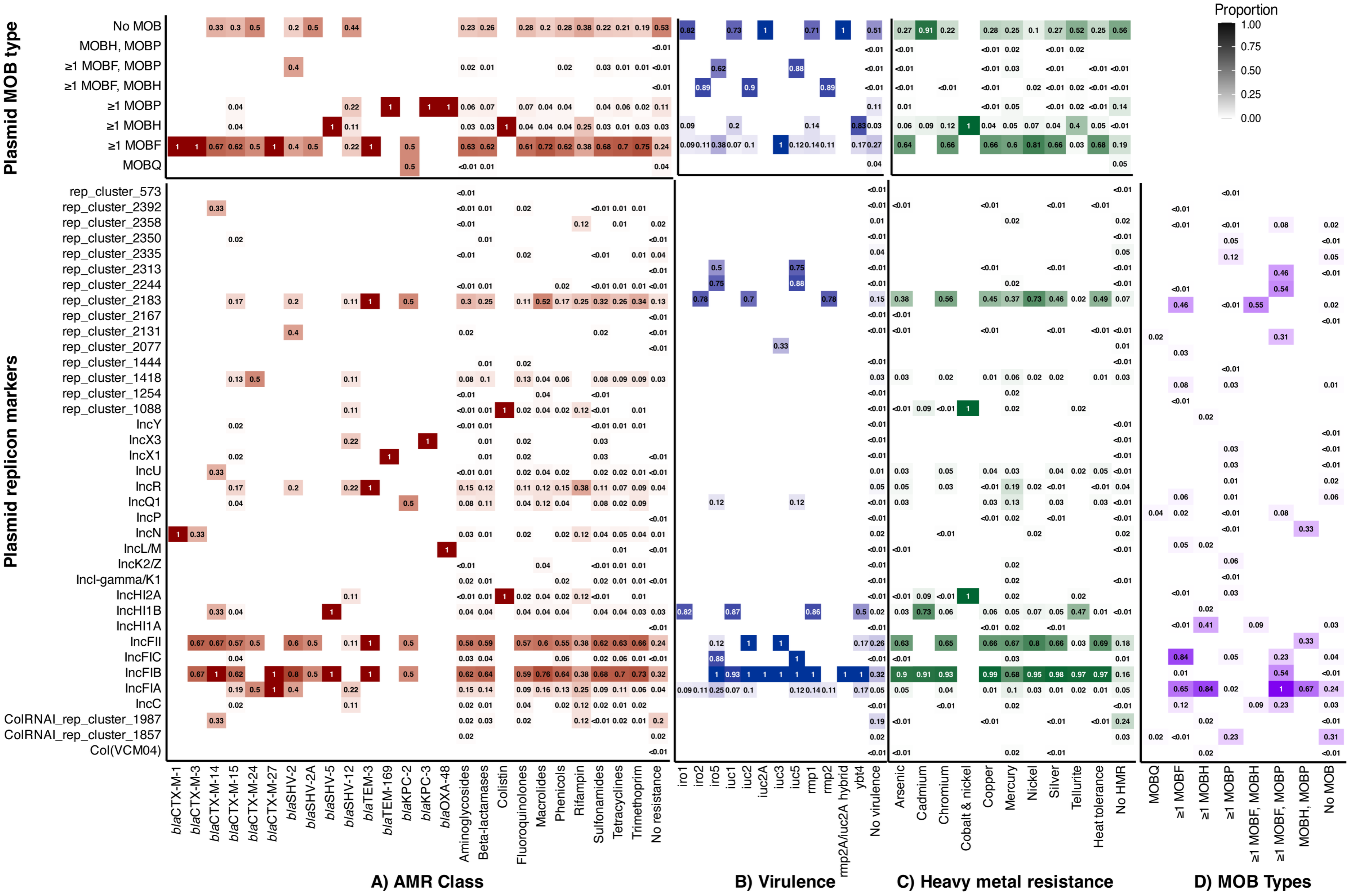

Fig. S5. Overlap of clinically relevant features with replicon and MOB types. Heatmap with proportion of **A)** antimicrobial resistance (AMR) genes (ESBLs and carbapenemases) or classes, **B)** virulence factors, **C)** heavy metal resistance (HMR) operons, or **D)** MOB types residing on plasmids with plasmid MOB types (top) and replicons (bottom). Not shown: plasmid MOB types and replicons that did not overlap with at least one AMR, virulence, or HMR category.

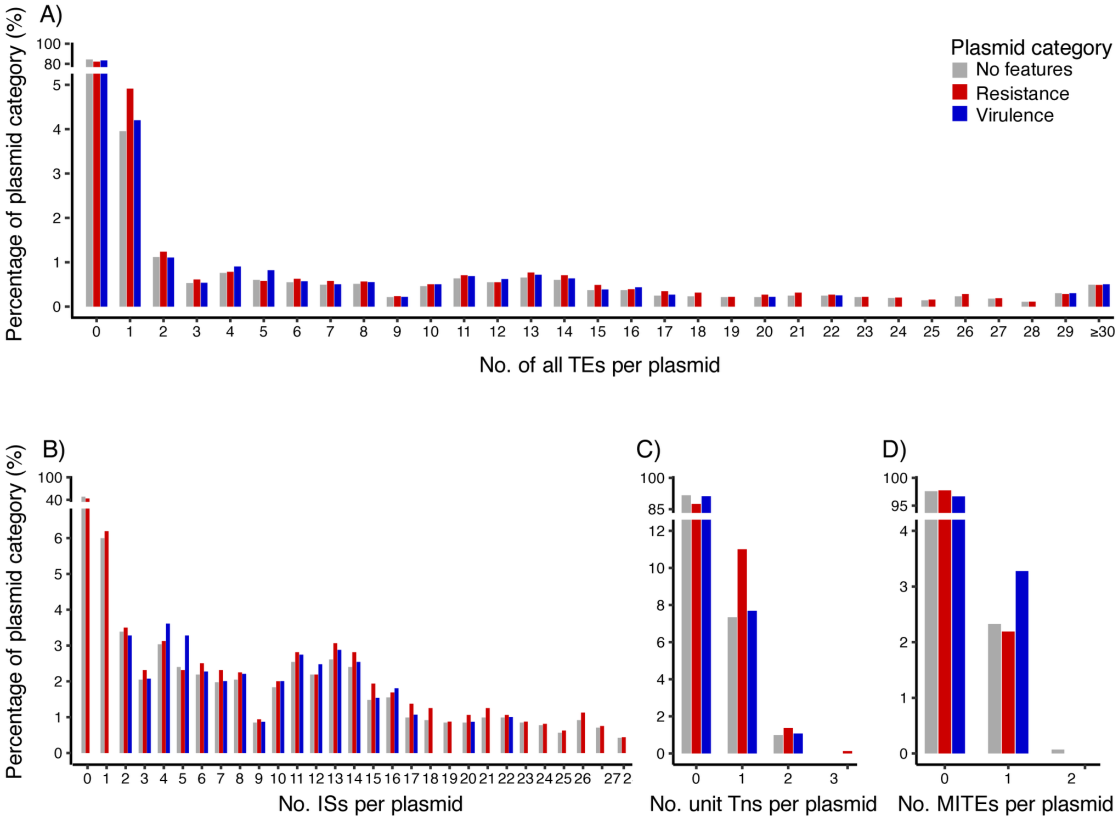

Fig. S6. Prevalence of transposable elements (TEs) across plasmid categories. Prevalence of **A)** all TE types, **B)** insertion sequences (ISs), **C)** unit transposons (Unit Tns), and **D)** miniature inverted-repeat transposable elements (MITEs) on antimicrobial resistance (AMR) (red), virulence (blue), and non-AMR/virulence (grey) plasmids.

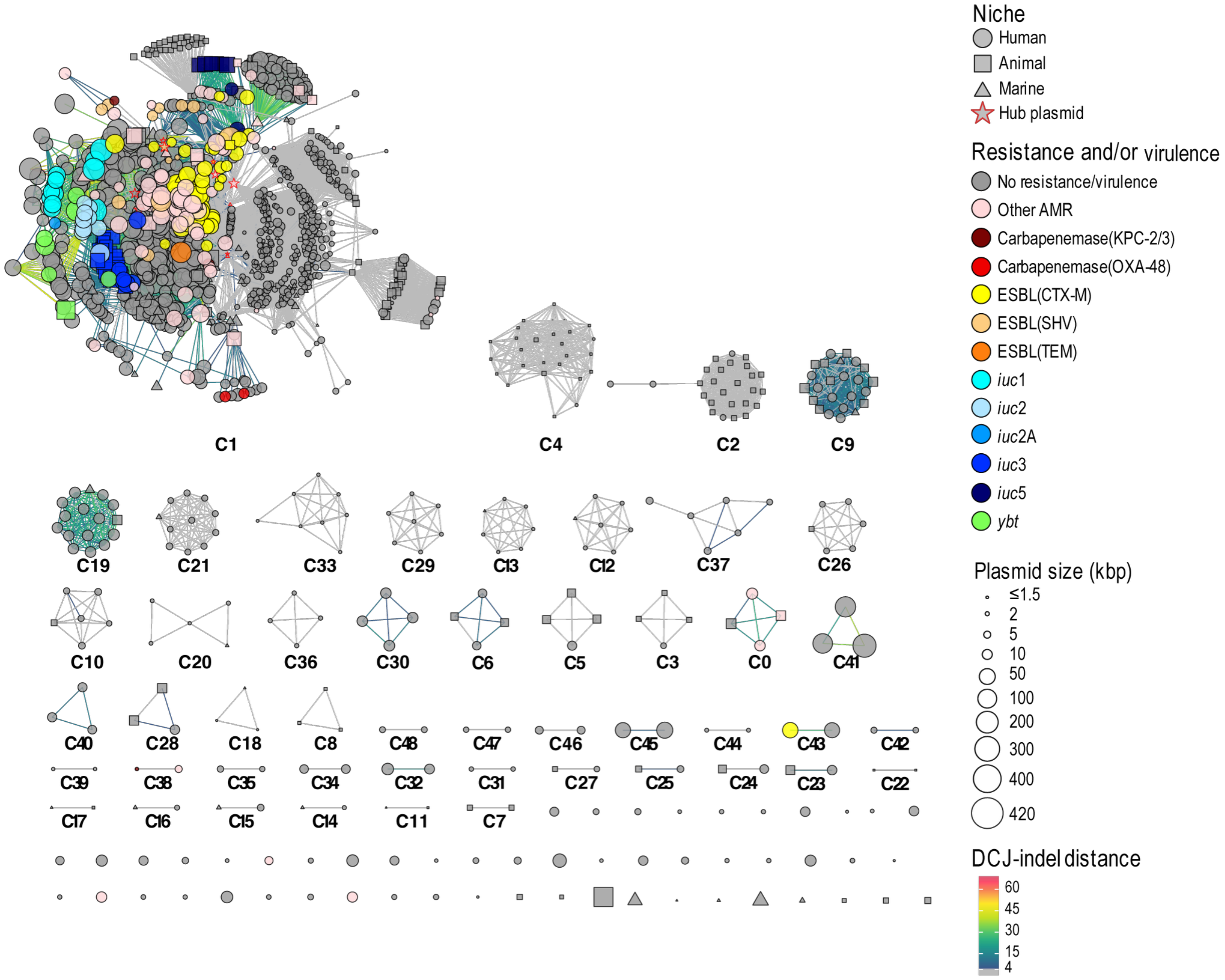

Fig. S7. Full Pling network graph (n=1415 plasmids) including all communities, clusters, singletons, and hub plasmids. Singletons are not labelled. Hub plasmids (n=13) were only present in the largest community (C1) and are shown as stars with red outlines. Of the 13 hub plasmids, 10 were found in the human niche, two of which encoded ESBLs, and three were found in the marine niche, none of which encoded AMR, HMR, or virulence.

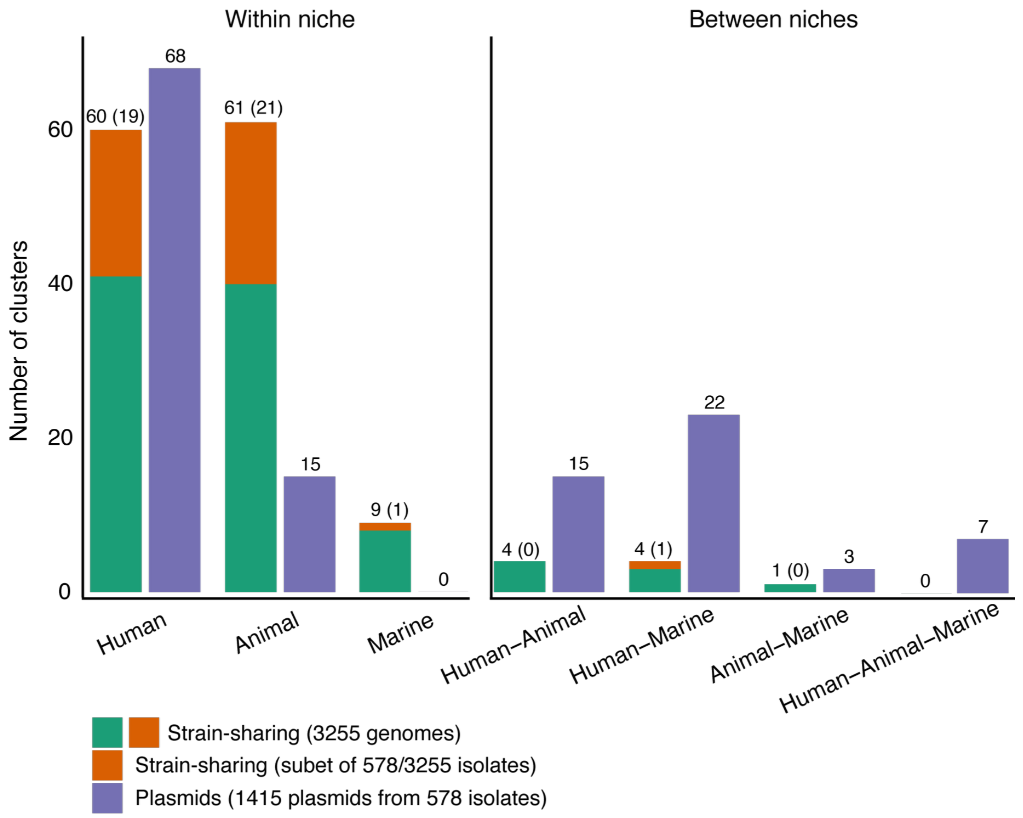

Fig. S8. Comparison of strain- and plasmid-sharing within and between niches. The number of strain-sharing clusters (defined as groups of isolates differing by ≤22 chromosomal SNPs) and plasmid-sharing clusters (defined as belonging to the same Pling cluster) is shown for both within-niche and between-niche comparisons. Because the plasmid analyses were restricted to a subset of 578 of 3255 isolates, the strain-sharing bars are coloured to indicate the contribution from this subset (orange), while the full bar height corresponds to counts from the entire isolate dataset. Total counts are shown above the bars, with values in parentheses indicating those from the hybrid-assembled subset.

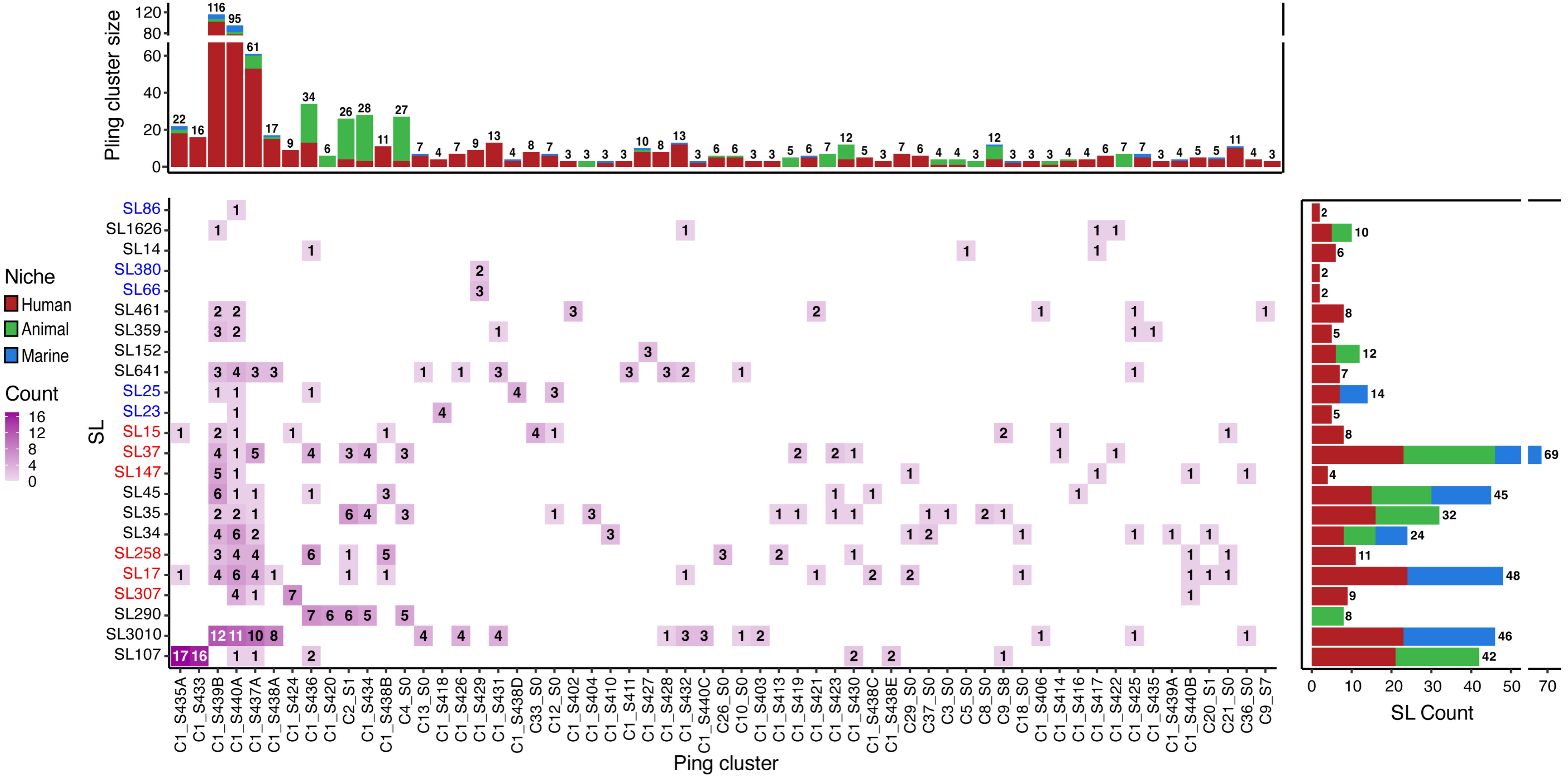

Fig. S9. Frequency of Pling cluster and SL overlap. Heatmap showing the frequency of each SL (y-axis) / Pling cluster (x-axis) overlap. SLs shown were represented by ≥5 isolates within the hybrid-assembled dataset, or SLs with <5 isolates but belonging to global MDR (red) or HV (blue) clonal groups; in all cases at least one isolate of that SL harboured ≥1 plasmid (n=23 SLs). Not shown: SLs present <5 times within the dataset and not part of global MDR or HV clonal groups, and SLs for which all isolates lacked plasmids (n=268 SLs). Marginal histograms indicate the number of plasmids per Pling cluster (top) or SL (right) present in each niche, coloured by niche.

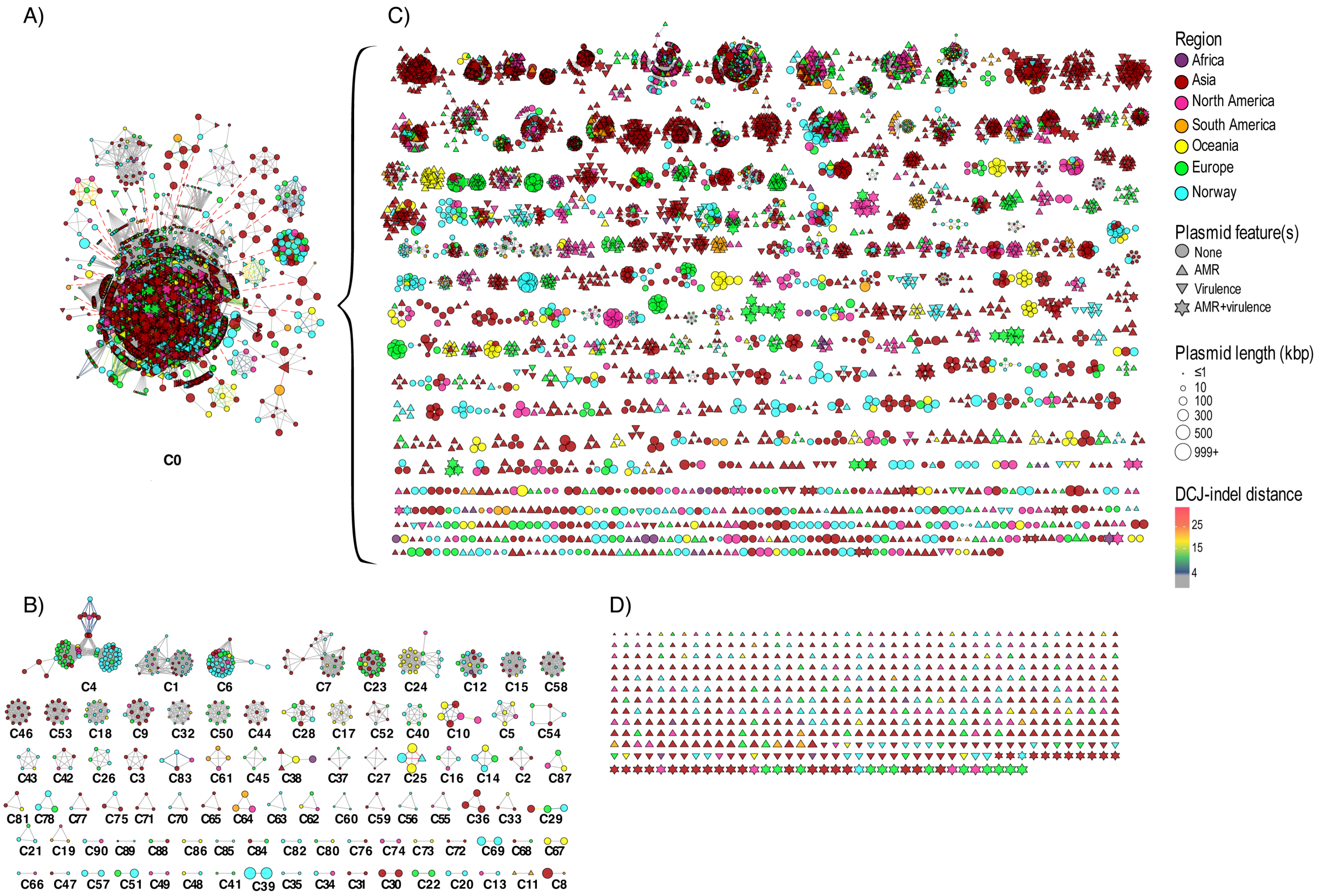

Fig. S10. Pling network graph of global and local plasmids. **A)** and **B)** Pling plot of communities within the global dataset, excluding 362 hub plasmids (n=9476 plasmids). Red dotted lines indicate connections that would have been made via hub plasmids. **C)** Subview of clusters within the largest Pling community (**A**) (n=7584 plasmids). **D)** Singletons harbouring antimicrobial resistance (AMR) genes and/or virulence factors (n=564/1544 singletons). Node shape indicates presence/absence of clinically relevant features, node size indicates plasmid length in kbp, node colour represents world region, as per inset legend. Edge colour indicates DCJ-indel distance between plasmids within the same community, where grey indicates ≤4 rearrangements between plasmids (i.e. plasmids within the same cluster). Not shown: singletons lacking AMR genes or virulence factors (n=980/1544 singletons) and hub plasmids (n=362; n=223 encoded AMR, n=3 encoded virulence, n=31 encoded AMR and virulence, n=105 lacked AMR or virulence factors).

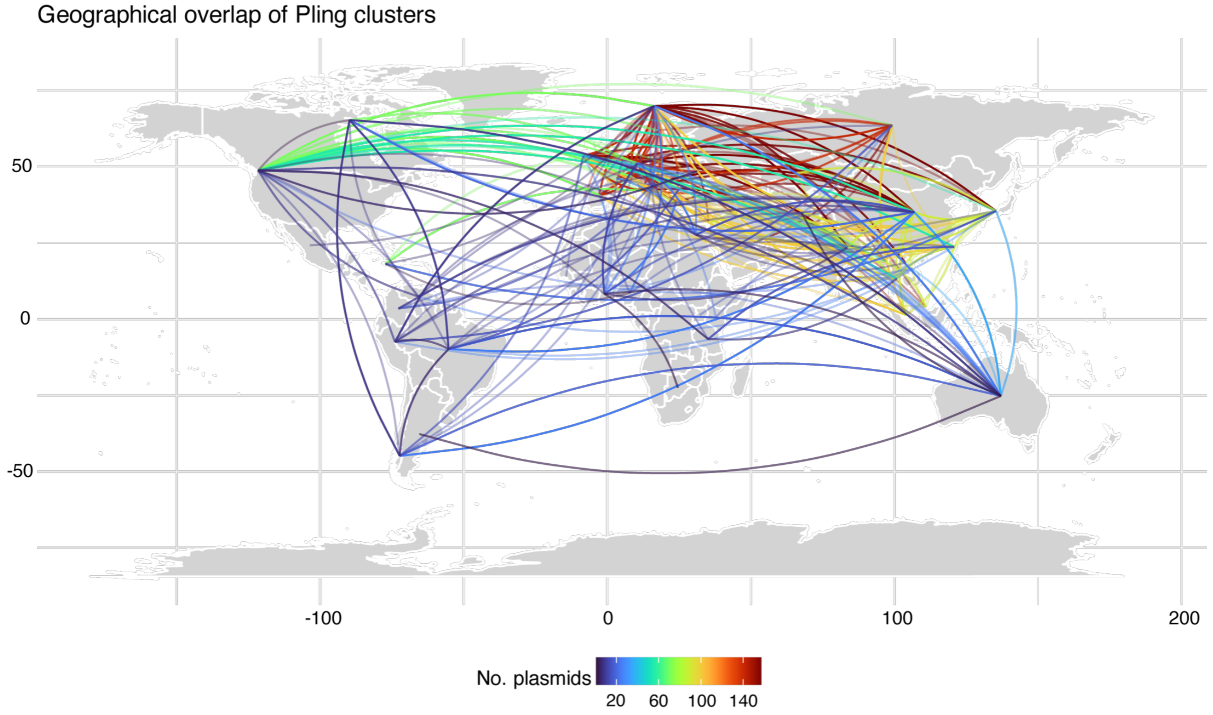

Fig. S11. Geographical overlap of plasmid clusters. Map showing plasmid clusters (n=325) shared across 65 countries and their geographic distribution. Colour scale indicates the number of plasmids shared between two locations.
